# Ex.50.T aptamer impairs tumor–stroma cross-talk in breast cancer by targeting gremlin-1

**DOI:** 10.1038/s41420-025-02363-6

**Published:** 2025-03-11

**Authors:** Cristina Quintavalle, Francesco Ingenito, Giuseppina Roscigno, Birlipta Pattanayak, Carla Lucia Esposito, Alessandra Affinito, Danilo Fiore, Gianluca Petrillo, Silvia Nuzzo, Bartolomeo Della Ventura, Federica D’Aria, Concetta Giancola, Stefania Mitola, Elisabetta Grillo, Marinella Pirozzi, Greta Donati, Francesco Saverio Di Leva, Luciana Marinelli, Zoran Minic, Francesca De Micco, Guglielmo Thomas, Maxim V. Berezovski, Gerolama Condorelli

**Affiliations:** 1 Institute of Endotypes in Oncology, Metabolism and Immunology “G. Salvatore” (IEOMI), Consiglio Nazionale delle Ricerche (CNR), Naples, Italy; 2 Department of Molecular Medicine and Medical Biotechnology, University of Naples Federico II, Naples, Italy; 3 AKA Biotech S.r.l, Naples, Italy; 4 IRCCS SYNLAB SDN, Naples, Italy; 5 Department of Physics “Ettore Pancini”, University of Naples Federico II, Naples, Italy; 6 Department of Pharmacy, University of Naples Federico II, Naples, Italy; 7 Department of Molecular and Translational Medicine, University of Brescia, Brescia, Italy; 8 Department of Chemistry and Biomolecular Sciences and John L. Holmes Mass Spectrometry Facility, University of Ottawa, Ottawa, ON Canada; 9 Mediterranea Cardiocentro, Naples, Italy

**Keywords:** Breast cancer, Nucleic-acid therapeutics, Drug development

## Abstract

The tumor microenvironment profoundly influences tumor complexity, particularly in breast cancer, where cancer-associated fibroblasts play pivotal roles in tumor progression and therapy resistance. Extracellular vesicles are involved in mediating communication within the TME, specifically highlighting their role in promoting the transformation of normal fibroblasts into cancer-associated fibroblasts. Recently, we identified an RNA aptamer, namely ex.50.T, that binds with remarkable affinity to extracellular vesicles shed from triple-negative breast cancer cells. Here, through in vitro assays and computational analyses, we demonstrate that the binding of ex.50.T to extracellular vesicles and parental breast cancer cells is mediated by recognition of gremlin-1 (GREM1), a bone morphogenic protein antagonist implicated in breast cancer aggressiveness and metastasis. Functionally, we uncover the role of ex.50.T as an innovative therapeutic agent in the process of tumor microenvironment re-modeling, impeding GREM1 signaling, blocking triple-negative breast cancer extracellular vesicles internalization in recipient cells, and counteracting the transformation of normal fibroblasts into cancer-associated fibroblasts. Altogether, our findings highlight ex.50.T as a novel therapeutical avenue for breast cancer and potentially other GREM1-dependent malignancies, offering insights into disrupting TME dynamics and enhancing cancer treatment strategies.

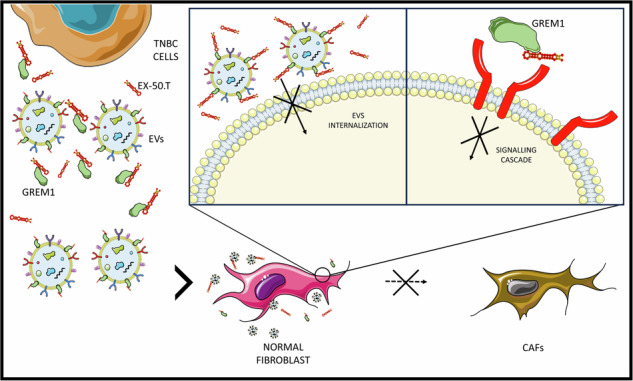

## Introduction

Tumor complexity is significantly influenced by the tumor microenvironment (TME) [1]. In breast cancer (BC), the TME consists of various cell types, including normal fibroblasts (NFs), immune cells, and endothelial cells, embedded in an extracellular matrix (ECM) [2]. Cancer-associated fibroblasts (CAFs) constitute a significant component of the TME, linked to invasive behavior and adverse prognoses in BC. In triple-negative BC (TNBC), CAFs assume a pivotal role in tumor progression [3, 4]. They actively contribute to remodeling the tumor environment by releasing proteins, such as collagen and fibronectin, leading to increased tissue rigidity that fosters tumor growth [5]. Moreover, CAFs support tumor expansion by promoting blood supply through the secretion of pro-angiogenic factors, like vascular endothelial growth factor (VEGF). They also orchestrate immune suppression by releasing factors that hinder the activity of T-immune cells, fostering an immunosuppressive environment that aids cancer cells in evading immune surveillance [6]. Additionally, CAFs have been implicated in chemoresistance, by forming protective barriers around cancer cells [4, 7].

The crosstalk between cells in the TME is partially mediated by extracellular vesicles (EVs) released from tumor cells. BC-derived EVs can influence neighboring cells, including NFs, by transferring bioactive molecules, such as proteins, nucleic acids, and lipids [8]. These EVs may also induce phenotypic changes in NFs, promoting their transformation into CAFs [9].

Therefore, strategies aimed at impairing EV release or uptake may be considered potential tools for disrupting the cancer-TME network and represent a potential avenue for cancer therapy. On this point, aptamers are short single-stranded oligonucleotide sequences generated by an in-vitro procedure named Systematic Evolution of Ligands by EXponential Enrichment (SELEX) [10, 11]. Similarly to antibodies, they can fold into three-dimensional structures, acquiring the ability to recognize specific target molecules [12]. The use of aptamers as blocking agents for EVs is an interesting avenue in research on potential therapeutic applications [13]. We recently described an RNA aptamer, named ex.50.T, that specifically recognizes BC-derived EVs [14].

Bone morphogenetic proteins (BMPs) are crucial in regulating bone and cartilage formation, maintenance, and repair by controlling various cellular processes [15]. Gremlin-1 (GREM1) is a BMP antagonist that directly binds to BMPs and operates as a TGF (transforming growth factor)-beta pathway inhibitor, preventing phosphorylation of SMAD1/5/8 and gene expression [15, 16]. Despite its traditional definition as a BMP antagonist, GREM1 is also reported to stimulate BMP-independent signaling pathways, including interactions with EGF (Epidermal Growth Factor) receptor [17], VEGFR2 [18, 19], heparan-sulfate proteoglycans [20], and FGFR1 [21] (Fibroblast Growth Factor Receptor 1). Interestingly, GREM1 is overexpressed in various carcinomas, such as those of the colon, lung, stomach, liver, and breast, influencing cancer cell proliferation, migration, and invasion [22, 23]. In BC, elevated GREM1 levels are associated with poor prognosis and the promotion of lung metastasis [24]. GREM1 also plays a role in activating CAFs and influencing their behavior within the TME [25, 26]. A recent study reported higher GREM1 levels in EVs from BC patients, raising questions about GREM1 transfer in the TME and to distant organs [27]. Overall, GREM1’s roles in cancer-related processes make it an intriguing protein for further investigation and a potential target for new therapeutic strategies.

In the present study, we demonstrate that ex.50.T specifically recognizes GREM1 also on EVs. Moreover, we explore the functional use of this aptamer in impairing EV-mediated crosstalk between TNBC cells and the TME, blocking EV-mediated transformation of NFs into CAFs. This study highlights ex.50.T as a novel candidate for further exploration as a potential therapeutic agent for BC and, potentially, other diseases in which GREM1 plays a substantial role.

## Results

### Ex.50.T specifically targets GREM1

**Fig. 1:**
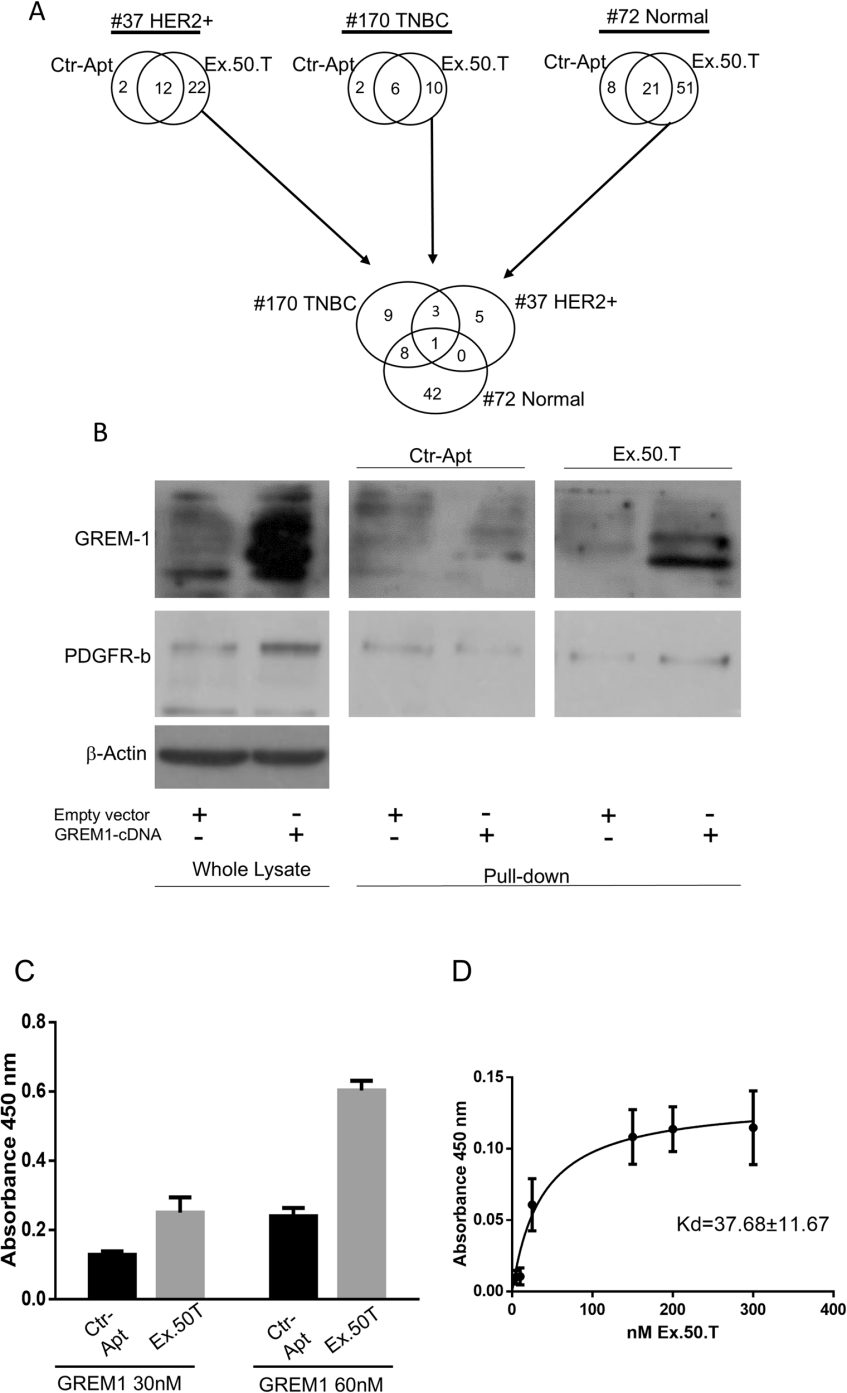
Ex.50.T specifically targets GREM1. **A** Workflow of proteomic analysis and Venn diagrams of the number of proteins for each group. **B** Western blot of ex.50.T pull-down proteins. PDGFR-β was used as loading control for pulled-down protein; β-actin was used as the loading control of whole lysate; and GREM1 was detected with an anti-GREM1 antibody (Cell Signaling Technology, # 4383S). **C** ELONA for Ex.50.T on 30 nMol and 60 nMol of recombinant protein. **D** Increasing concentrations of ex.50.T or CtrlApt were incubated on plates uncoated or coated with GREM1 recombinant protein. Specific binding of ex.50.T was determined by subtracting the values obtained with CtrlApt and reported as Kd ± SE.

To identify the target of the aptamer ex.50.T, we implemented a pull-down assay with a biotinylated ex.50.T and a scrambled (CtrlApt), unrelated sequence on protein extracts of EVs shed from patient-derived cell lines of a fibroadenoma (Pt.72) and two BCs: a HER2^+^ BC (Pt.37) and a TNBC (Pt.170). We selected these because ex.50.T bound EVs shed from Pt.37 and Pt.170 but not from Pt.72 [14]. Eluted proteins were analyzed by liquid chromatography-mass spectrometry. After subtraction of CtrlApt-bound proteins, 51 proteins were identified as unique to Pt.72, 21 proteins to Pt.37, and 9 proteins to Pt.170 (Supplementary Table 1). Putative targets of ex.50.T were identified by focusing on the proteins pulled-down from samples from Pt.37 and Pt.170 but not from Pt.72, with a *q*-value < 0.001. Three proteins met the criteria: CCDC80 (coiled-coil domain containing 80), GREM1, and DSP (desmoplakin) (Fig. 1A and Supplementary Table 2). Since DSP has been reported to be downregulated in the most aggressive forms of breast cancer [28] and CCDC80 has been primarily characterized as a tumor suppressor gene [29], we decided to focus on GREM1 that was recently reported to promote breast cancer metastasis [24], and has been found on blood-derived exosomes of BC patients [27].

We examined the pulled-down proteins by western blotting with an anti-GREM1 antibody to validate the interaction between ex.50.T and GREM1, confirming the aptamer’s ability to directly interact with the protein (Fig. 1B). An enzyme-linked oligonucleotide assay (ELONA) with a recombinant GREM1 protein further confirmed a direct interaction between ex.50.T and GREM1 (Fig. 1C) and allowed us to determine the apparent dissociation constant of ex.50.T for the recombinant GREM1 protein (Kd±SE of 37.68 ± 11.67) (Fig. 1D). To further evaluate sequence specificity and affinity for GREM1, we conducted Surface plasmon resonance (SPR) experiments. The Kd value of ex.50.T. and GREM1, obtained from these experiments, is in perfect agreement with that obtained from ELONA assay (Table 1 and Supplementary Fig. 1A). Altogether, these findings indicated that ex.50.T directly binds GREM1 with high affinity.

**Table 1:** Kinetics and thermodynamic parameters for the interaction of GREM1 with Ex.50.T aptamer.

	*k*_on_ (M^−1^ s^−1^)^a^	*k*_off_ (s^−1^)^a^	*K*_D_ (nM)^b^
Ex.50.T aptamer	3.39 × 10^4^	1.58 × 10^−3^	46.7

^a^Errors were within 5%.

^b^Errors were within 10%.

### Modeling of ex.50.T–GREM1 binding

**Fig. 2:**
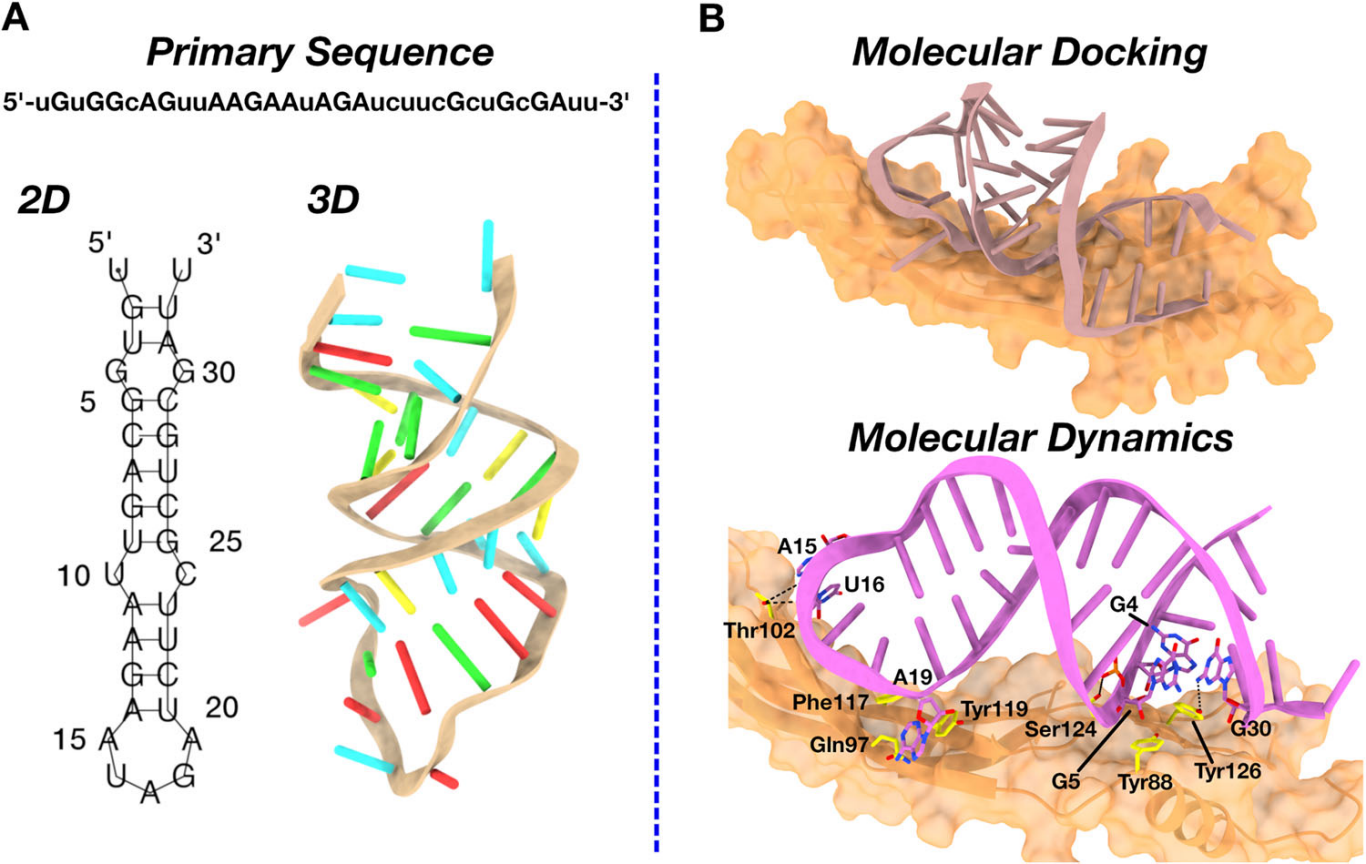
Modeling of ex.50.T on GREM1. **A** Schematic representation of ex.50.T in silico modeling from the primary sequence. The minimum free energy 2D structure predicted by 5 programs (RNAfold, RNAstructure, Mfold, pKiss, MXfold2) is shown with line segments between nucleotides representing base pairings. The 3D structure obtained by RNAComposer is shown as a cartoon, with nucleotides highlighted as stubs (color code: A, red; C, yellow; G, green; U, cyan). **B** Schematic representation of the ex.50.T-GREM1 complex from consensus docking according to the procedure described in the “Materials and methods” section. GREM1 is represented in orange surface and the aptamer as a stub (top panel). Ex.50.T interactions with GREM1 as predicted by MD simulations (bottom panel). Ex.50.T nucleotides and GREM1 residues mainly involved in the aptamer-protein interaction are highlighted as sticks. Hydrogen bonds are depicted as dashed black lines. For the sake of clarity, hydrogen atoms are omitted and only the most conserved interactions along the MD simulations are highlighted.

To investigate at atomic-resolution level the interaction between ex.50.T and GREM1, we implemented a combined bioinformatics and biosimulation approach. First, we envisioned the 2D structure of ex.50.T, employing five different bioinformatics tools relying either on a thermodynamic model [30] (RNAfold^46^, RNAstructure [31], Mfold [32]), on the inclusion of pseudoknots formation (pKiss [33]), or a deep-learning approach (MXfold2 [34]) (see Material and Methods in Supplementary Information for details). The predictions of these models converged on an identical secondary structure, featuring a stem composed of 11 canonical base pairs, 2 internal loops, and 1 terminal loop (Fig. 2A).

The high redundancy among the employed programs prompted us to use the identified 2D structure as the starting point to bioinformatically predict the 3D conformation of ex.50.T. To this aim, the 2D model was processed by RNAComposer [35], a widely used software to predict reliable 3D aptamer structures (see Methods) [36–38]. The resulting 3D structure (Fig. 2A) was then submitted to a *consensus* docking computational procedure in which five software programs performing either rigid (ZDock [39], HDock [40], PatchDock [41], NPDock [42]) or semi-flexible (HADDOCK [43]) molecular docking were used to provide the initial model of ex.50.T’s interaction with GREM1. The ten lowest energy RNA–protein complexes provided by each software were clustered together, and the centroid structure of the most populated cluster was chosen for subsequent analysis. In this structure, the aptamer bound GREM1 parallel to the protein’s anti-parallel β-sheets (Fig. 2B).

To evaluate the reliability of the predicted docking pose, we performed a 1.5 μs all-atom molecular dynamics (MD) calculation in explicit water, considering complete RNA and protein flexibility, as well as the solvent and ion effects. In the first 0.25 μs of the simulation, the aptamer underwent an initial rearrangement, as highlighted by root-mean-square deviation analysis of the RNA heavy atoms (Supplementary Fig. 1C), to optimize the interactions network while retaining its overall orientation (Fig. 2B and Supplementary Fig. 1B). Indeed, the MD predicted pose established tight interactions with GREM1 in correspondence with multiple protein regions, which were largely conserved for the entire simulation (Fig. 2B). In detail, retained π-π stacking interactions involved Tyr88 and Tyr126 with G5 and G4, respectively (Fig. 2B and Supplementary Fig. 2), while hydrogen bonds were established between Ser124 and G5 and Tyr126 and G30 (Fig. 2B and Supplementary Fig. 2). An additional hydrogen bond was formed by Thr102 with U16, whereas the same amino acid implemented a more labile interaction with A15 (Fig. 2B and Supplementary Fig. 2). Further interactions were established by A19 with Gln97, Phe117, and Tyr119, with the latter being the most conserved along the simulation time (Fig. 2B and Supplementary Fig. 2). Notably, A15, U16, and A19 belong to the hairpin loop that, besides the less constrained terminal nucleotides, was the most flexible region of ex.50.T, as testified by root-mean-square fluctuation analysis showing values above 2 Å for these residues (Supplementary Fig. 1D). Altogether, these computational studies identified the aptamer regions mostly involved in binding to GREM1, paving the way for future modifications aimed at improving target affinity, selectivity, and in vivo stability.

### Ex.50.T binds GREM1 on BC cells

**Fig. 3:**
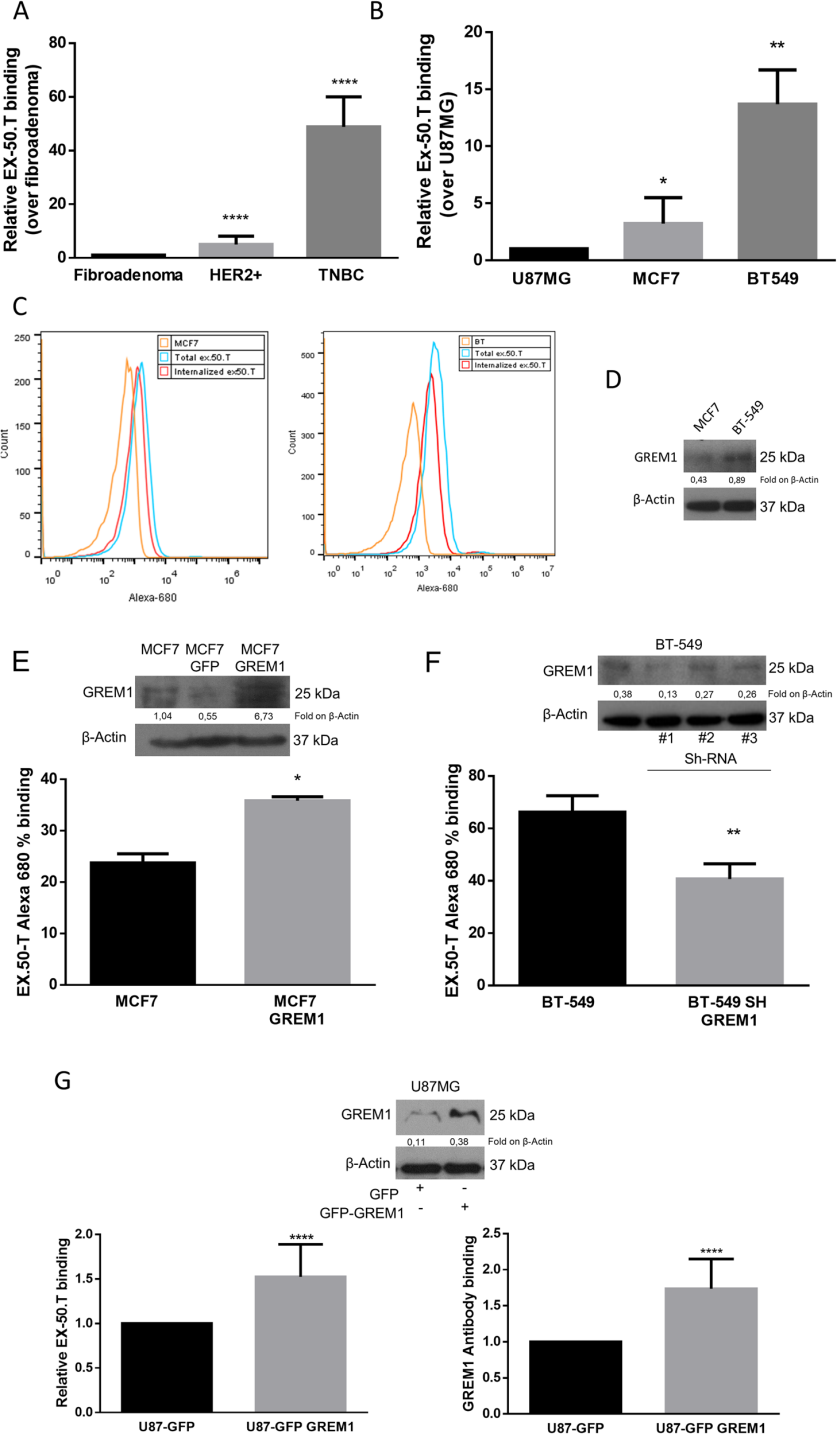
Binding analysis of ex.50.T on continuous cell lines and GREM1-modulated cell lines. **A** The binding ability of ex.50.T on parental cells of EVs used for exosome SELEX was analyzed by qPCR. Data are mean percentage ± SD over control (fibroadenoma) of two biological replicates. **B** qPCR analysis of ex.50.T binding on BC continuous cell lines; U87MG cells were used as control cell line. Data are mean percentage ± SD over control of two biological replicates. **C** Flow cytometry analysis of Alexa-680–ex.50.T binding on PBS-washed MCF7 and BT-549 cells (Total) and high-salt-treated MCF7 and BT-549 cells (Internalized). **D** Western blot of GREM1 (R&D Systems, # AF956) in MCF7 and BT-549 cell lines. β-actin was used as a loading control. **E** FACS analysis of ex.50.T in GREM1-overexpressing MCF7 cells (MCF7 24% vs. MCF7 GFP 36%). Data are mean percentage ± SD over control of two independent experiments in three replicates. Western blot analysis was used for overexpression control of GREM1 (Santa Cruz Biotechnology, # sc-515877); β-actin was used as loading control. **F** FACS analysis of ex.50.T in BT-549 cells after interference with GREM1. Data are mean percentage ± SD over control (BT-549) of all sh-GREM1 interfered points of two independent experiments in three replicates. Western blot analysis was used for down-modulation control of GREM1 (R&D Systems, # AF956); β-actin was used as loading control. **G** ELISA with GREM1 antibody (sc-515877) (right) and biotinylated ex.50.T ELONA (left) on total protein lysate of U87MG cells overexpressing GREM1. Data are mean percentage ± SD over control U87MG cells of three independent experiments in triplicates. Western blot analysis was used for overexpression control of GREM1 (R&D Systems, # AF956); β-actin was used as loading control. *p < 0.05; **p < 0.01; ***p < 0.001.

Having confirmed that ex.50.T binds EVs of patient-derived BC cell lines of (Pt.37 and Pt.170) but not fibroadenoma (Pt.72), we assessed the aptamer’s capacity to bind parental cell lines. There was an increased binding to tumoral cell lines compared to the fibroadenoma one (Fig. 3A). Moreover, as reported for ex.50.T and EVs [14], real-time PCR revealed that the aptamer interacted with cells of the continuous BC cell lines BT-549 and MCF7 more efficiently than with U87MG cells, a glioblastoma cell line (Fig. 3B). Flow cytometry analysis with ex.50.T labeled with Alexa 680 fluorochrome revealed that the aptamer had a higher binding capacity for BT-549 cells than for MCF7 cells (Fig. 3C). Additionally, ex.50.T was readily internalized by BT-549 and MCF7 cells, reaching ~54% and ~44% cell internalization, respectively, following 30 min of incubation (Fig. 3C and Supplementary Fig. 3A). Western blotting revealed that BT-549 cells expressed more GREM1 than did the MCF7 cells, indicating that the increased binding capacity of the former for ex.50.T is possibly linked to the higher level of this protein (Fig. 3D).

To assess the specificity of ex.50.T binding, we analyzed binding to MCF7 cells, which normally express a low level of GREM1, modified to stably overexpress human GREM1 (Fig. 3E and Supplementary Fig. 3B). As expected, cytofluorimetric analysis showed that ex.50.T bound to the modified MCF7 cells to a greater extent than to parental cells (Fig. 3E). In contrast, down-regulation of GREM1 in BT-549 cells, mediated by a short-hairpin (sh)-GREM1 lentiviral construct (Fig. 3F and Supplementary Fig. 3C) or by GREM1 siRNA (Supplementary Fig. 3D), reduced aptamer binding.

We then compared the abilities of ex.50.T and a GREM1 antibody to recognize GREM1 in lysates from a U87MG cell line overexpressing GREM1. ELONA/ELISA revealed that the two had comparable binding abilities (Fig. 3G). Also, ex.50.T and the antibody had similar abilities to recognize a GREM1 recombinant protein (Supplementary Fig. 3E).

These findings suggest that ex.50.T recognizes GREM1 on the cell surface, so may be a promising tool for the specific delivery of therapeutic cargo to GREM1*-*overexpressing cells.

### Ex.50.T inhibits internalization of TNBC-derived EVs in NFs

**Fig. 4:**
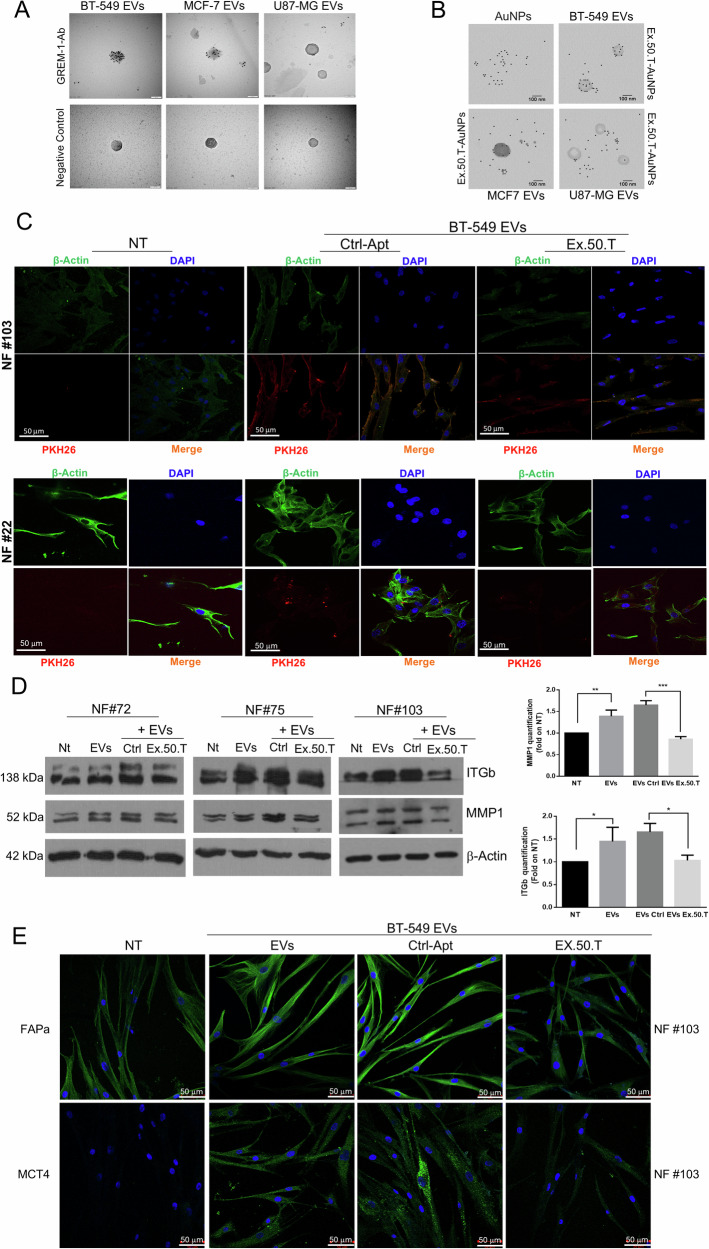
GREM1 expression on EVs and Ex.50.T-mediated impairment of BT-549-derived EV uptake and action in NFs. **A** Transmission electron micrographs of isolated EVs from BC and glioblastoma cell lines. Each dot is the binding of the primary antibody to GREM1. The GREM1 level appears highest in BT-549 EVs, followed by MCF7 EVs, and finally U87MG EVs. Scale bars = 100 nm. **B** Transmission electron micrographs of isolated EVs from BC and glioblastoma cell lines. Each dot is the binding of AuNPs functionalized ex.50.T aptamer. The GREM1 level appears higher in BT-549 EVs, compared to MCF7 EVs and U87MG EVs. Scale bars = 100 nm. **C** Representative images from confocal microscopy of NFs (NFs) from two patients, exposed to PKH26-labeled, BT-549-derived EVs, pre-incubated with either ex.50.T aptamer or CtrlApt. The image shows reduced EV internalization compared to CtrlApt, as evinced by the decreased merged signal (orange). PKH-26 alone was used as negative control (NT). All images were captured at the same settings, enabling direct comparison of staining patterns. NFs were stained using DAPI (blue) and ALEXA488-conjugated anti-β-actin antibody (green), respectively for nuclei and cytoskeleton detection. Magnification 63x. **D** Western blots on three different NFs for MMP1 and ITGb1 upon treatment of BT-549-derived-EVs, CtrlApt, and ex.50.T, compared to untreated NFs. β-actin was used as loading control. Right panel densitometric analysis of western blot quantification. Data are presented as mean value ± SD over control (NT) of the three biological replicates. **E** Representative images from confocal microscopy of FAPa and MCT4 (Green, FITC) immunostaining in NFs, exposed to BT-549-derived EVs, pre-incubated with either ex.50.T aptamer or control aptamer (CtrlApt). Nuclei were stained with DAPI (blu).

GREM1 is present on BC-derived EVs [27]. Analysis of GREM1 expression on the surface of BC cell EVs by immuno-electron microscopy using a GREM1 antibody confirmed higher expression of GREM1 on BT-549 EVs compared to MCF7 EVs and, interestingly, lower expression on glioblastoma U87MG-derived EVs compared to the former two (Fig. 4A). Moreover, TEM analysis revealed that BT-549 EVs samples were surrounded by a significantly higher number of functionalized AuNPs (fAuNPs)-ex-50.T compared to MCF7 and U87MG samples, confirming the same epitope variability between analyzed samples (Fig. 4B). These findings support our model explaining the preferential targeting of BT-549 and MCF7 EVs by the ex-50.T [14].

The reciprocal interaction between breast cancer cells and CAFs is mediated in part by GREM1 [26]. Since ex.50.T can bind BC-derived EVs in a GREM1-dependent manner, we investigated its potential to inhibit EV-mediated crosstalk between BC cells and NFs, the major component of the breast cancer TME [3, 44]. To this aim, we first isolated and characterized by nanoparticle tracking analysis (NTA) and western blotting BT-549 cell EVs, which highly express GREM1, detecting a peak around 200 nm in NTA analysis and the expression of Alix, CD81, and CD9, but not calnexin, by western blotting (Supplementary Fig. 4A). Then, we incubated stromal NFs derived from fibroadenoma specimens (NF#22, NF#72, NF#103) with PKH-26-labeled BT-549 EVs, in the presence or not of ex.50.T. Immunofluorescence and cytofluorimetric assays revealed that BT-549-derived EVs were efficiently taken up by NFs (Supplementary Fig. 4C, D), and that the presence of ex.50.T impaired EV internalization (Fig. 4B and Supplementary Fig. 4D).

### Ex.50.T inhibits fibroblast activation induced by TNBC-derived EVs

EVs originating from TNBC cells can impact nearby cells, such as NFs, triggering alterations in their characteristics and leading to their conversion into CAFs [45, 46]. To investigate whether ex.50.T antagonizes this transformation, we incubated NFs (#22, #72, #75, #103) with BT-549-derived EVs, with BT-549-derived EVs pre-incubated with ex.50.T, or with BT-549-derived EVs pre-incubated with a control aptamer, evaluating changes in molecular markers associated with the CAF phenotype. Considering that aggressiveness and metastasis formation result from changes in the composition of the ECM and cell–ECM interactions orchestrated by the collaborative influence of integrins and matrix metalloproteinases (MMPs) [47], we explored whether tumoral EVs altered the expression of these markers. We observed an upregulation of MMPs, specifically MMP1, as well as β1 integrins by Western blot (Fig. 4D, E) and of MCT4, FAPα, and β1 integrins in NFs by immunofluorescence (Fig. 4E and Supplementary Fig. 5A) when exposed to BT-549-derived EVs. Notably, the presence of ex.50.T reversed this effect. We then employed medium from BC patient-derived organoids to stimulate the transformation of NFs into CAFs and assess any inhibition by the aptamer. The conditioned medium effectively triggered the conversion of NFs to CAFs, a process hindered by the presence of ex.50.T (Supplementary Fig. 5B).

**Fig. 5:**
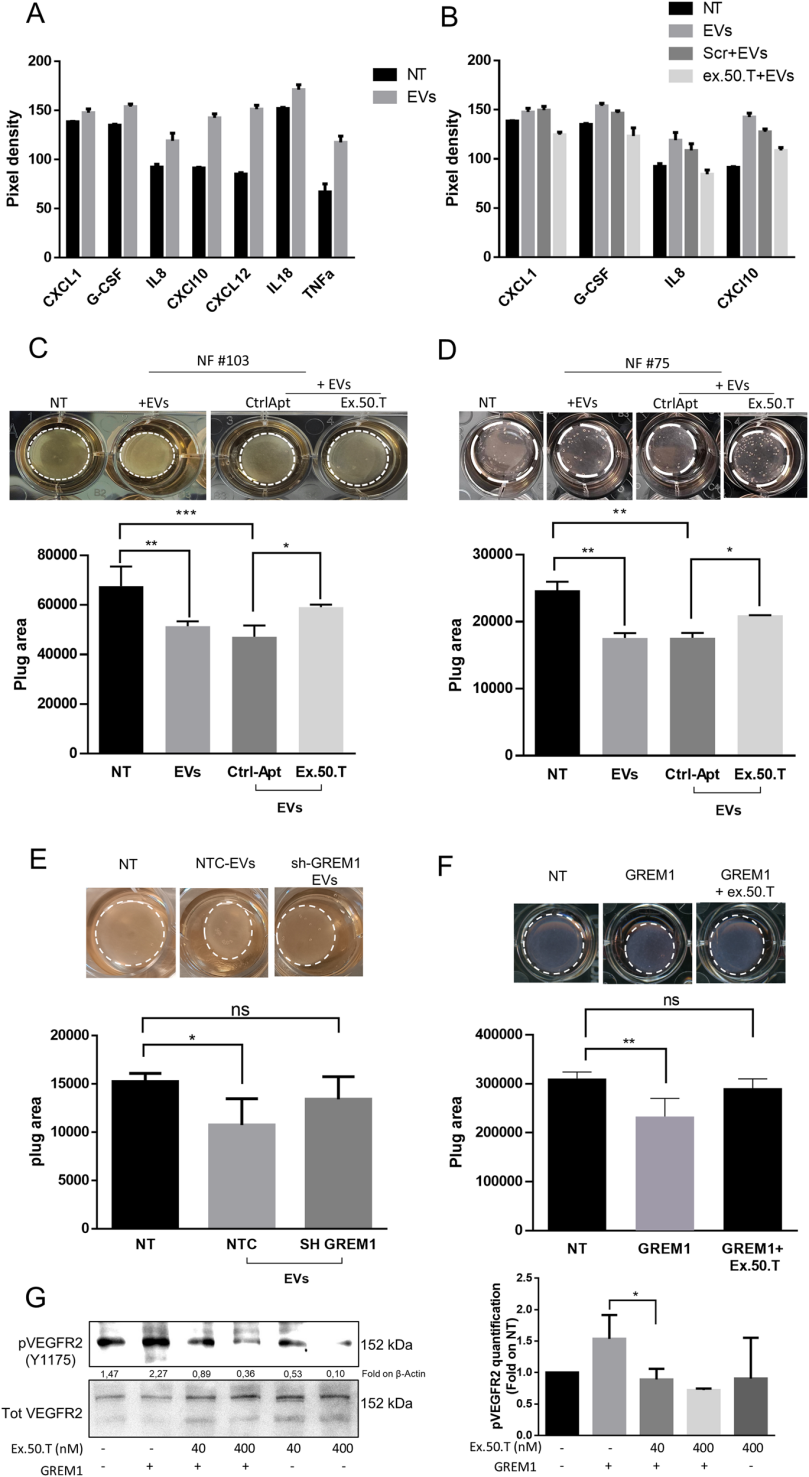
Ex.50.T reduces fibroblast-mediated extracellular matrix remodeling. **A** Proteome profile Cytokine array analysis of media of NFs treated with EVs alone or in combination with control aptamer or ex.50.T (**B**). Data are reported as pixel intensity measured with ImageJ. **C**, **D** Collagen contraction assay for two patient-derived NFs (top). Histograms (bottom) of collagen plug areas measured with ImageJ. Data are reported as mean + SD of two biological duplicates of two technical replicates. **E** Collagen contraction assay. Shown are representative pictures of collagen plugs containing NFs treated with EVs from BT-549 cells stably expressing sh-RNA GREM1 (SH-GREM) and a sh-scrambled sequence (NTC). Histograms (bottom) of collagen plug areas, measured with ImageJ. Standard deviations were calculated on replicates from three independent experiments performed with patient-derived NF cells (pt. #72, #75). **F** Collagen contraction assay. Shown are representative pictures of collagen plugs containing NFs treated with gremlin 1 recombinant protein with or without 500 nMol of ex.50.T (top). Histogram (bottom) of collagen plug areas measured with ImageJ. Standard deviations were calculated on replicates from three independent experiments performed with one patient-derived NF cells (pt. #22). **G** Serum-starved HUVECs were stimulated with 16 nM of GREM1 for 10 min in the presence or the absence of increasing doses of ex.50.T. Phospho-VEGFR2 (Y1175) and total VEGFR2 (loading control) levels were assessed by Western blot in cell lysates. On the left densitometric quantification of three independent WB experiments. *p < 0.05; **p < 0.01; ***p < 0.001; ****p < 0.001.

Furthermore, we observed an elevation of established known CAF-secreted cytokines in the culture medium of NF (#103) following treatment with EVs. Specifically, we detected increased levels of CXCL1, G-CSF, IL-8, CXCL10, and CXCL12 (Fig. 5A and Supplementary Fig. 6). Notably, the secretion of CXCL1, G-CSF, IL-8, and CXCL10 was significantly reduced in the presence of ex.50.T (Fig. 5B and Supplementary Fig. 6).

In conclusion, ex.50.T disrupts the EVs-mediated activation of NFs to CAFs, highlighting its potential in modulating the aggressive and metastatic features associated with alterations in the TME.

### Ex.50.T hinders the migratory and invasive properties induced in CAFs by TNBC EVs

To further corroborate the effect of ex.50.T on fibroblast activation evoked by BC EVs, NFs (#75 and #103) were exposed to BT-549 EVs: this resulted in a notable reduction in collagen plug area compared to untreated NFs (Fig. 5C, D). Interestingly, the collagen plug area was widened when NFs were exposed to EVs that had been pre-incubated with ex.50.T, indicating there was hindrance of fibroblast contractile activity (Fig. 5C, D).

GREM1 has been reported to promote CAF activation and regulate their behavior [26]. When NFs were exposed to EVs from BT-549 with GREM1 interference, their ability to induce collagen contraction decreased (Fig. 5E). Conversely, the exposure to recombinant GREM1 led to collagen gel contraction by NFs, an effect that was reversed by co-treatment with ex.50.T (Fig. 5F). Furthermore, GREM1 is an agonist of VEGFR2, determining its phosphorylation in HUVECs (human umbilical vein endothelial cells) [18, 48]. Therefore, we exposed HUVECs to GREM1 and ex.50.T. The aptamer reverted GREM1-induced VEGFR2 phosphorylation in a dose-dependent manner (Fig. 5G).

**Fig. 6:**
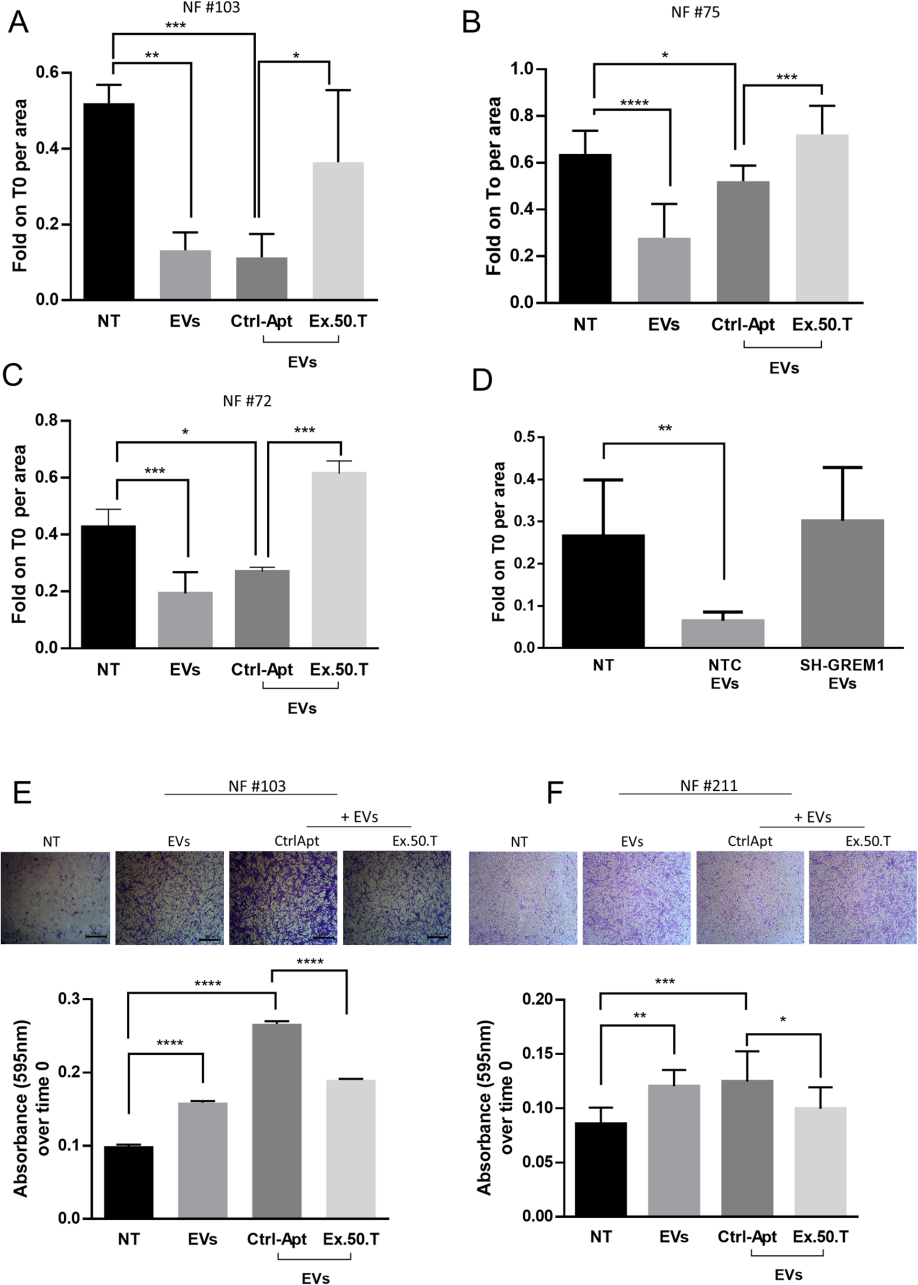
BC-derived EVs and ex.50.T aptamer affects NF motility. **A**–**C** Histograms of measured area upon wound healing of NFs treated with EVs alone or pre-incubated with ex.50.T aptamer compared to the CtrlApt. Wound healing areas at 24 h were normalized on the t0 areas and were measured in at least two independent wound sites. Standard deviations were calculated on replicates from three independent experiments performed for each patient. **D** Histograms of measured area upon wound healing of NFs treated with EVs from BT-549 cells stably expressing sh-RNA GREM1 (SH-GREM) and a sh-scrambled sequence (NTC). Wound healing areas at 24 h were normalized on the t0 areas. Standard deviations were calculated on replicates from three independent experiments performed on NFs. **E**, **F** Representative micrographs (top) of NFs migrated through the transwell upon 24 h of treatment, stained with crystal violet. Histograms (bottom) represent the absorbance values of crystal violet (595 nm) eluted from migrated cells, in NFs treated with BT549-derived EVs pre-incubated with ex-50.T or the CtrlApt. Standard deviations were measured in two independent experiments. *p < 0.05; **p < 0.01; ***p < 0.001; ****p < 0.0001.

To further characterize the inhibitory effect of ex.50.T on the EV-mediated conversion of NFs to CAFs, we conducted two different assays to assess migratory phenotype: a wound healing assay and a transwell migration assay. For the former, NFs were plated and scratches deliberately created on the wells; subsequently, cells were exposed to BT-549-derived EVs, to EVs pre-incubated with ex-50.T, or to a control aptamer. EVs enhanced the wound closure, and the phenotype was mitigated in cells exposed to the ex.50.T-treated EVs compared to the control aptamer-treated EVs (Fig. 6A–C and Supplementary Fig. 7A–C). Interestingly BT-549 EVs interfered for GREM1 expression were unable to promote wound closure (Fig. 6D and Supplementary Fig. 7D). To corroborate these findings, NFs (#211, #103) were seeded in the upper chamber of a transwell with serum-free medium to induce cell migration through a nutrient gradient. Exposure to BT-549-derived EVs increased the percentage of migrating fibroblasts, whereas cells exposed to EVs pre-incubated with the aptamer exhibited a 30% reduction compared to the control (Fig. 6E, F).

These findings demonstrate that the heightened migratory ability induced by BT-549-derived EVs in NFs is significantly reduced in the presence of the ex.50.T, suggesting the potential utility of the aptamer as a tool to mitigate the pro-migratory effects on CAFs mediated by BC EVs.

## Discussion

Due to the prevalence and complexity of BC, it is imperative to develop novel therapeutic strategies that address the limitations of conventional antitumoral treatments, including their low specificity, limited efficacy, and adverse side effects. Targeted therapies would better address the unique characteristics of each patient’s tumor, ultimately improving treatment outcomes and boosting quality of life [49]. For example, hormone therapies and HER2-targeting drugs have resulted inadequate for TNBC, a cancer lacking estrogen/progesterone receptors and with excess HER2 proteins. Consequently, there is a critical need for innovative therapies to enhance survival rates and outcomes for TNBC patients [50, 51].

Moreover, treatment efficacy is no longer solely reliant on cancer cells but also on the other cellular constituents of the TME. EVs—small vesicles secreted by various cell types—facilitate communication between tumor cells and the surrounding microenvironment by exchanging proteins, lipids, and nucleic acids [52]. EVs derived from BC cells can impact neighboring cells, notably fibroblasts, by transferring bioactive substances, like proteins, nucleic acids, and lipids. These EVs can prompt phenotypic alterations in NFs, potentially leading to their transition into CAFs [9], cells with a pivotal role in reshaping the TME and fostering tumor proliferation, invasion, and metastasis [3, 53].

Cancer-derived EVs can stimulate fibroblasts to become highly invasive [54]. These activated fibroblasts exhibit increased expression of proteins crucial for membrane protrusion and invasion. Additionally, they secrete elevated levels of matrix-degrading enzymes like MMP11 and ADAM10, facilitating their penetration through the extracellular matrix [55].

The specific mechanisms through which BC EVs trigger fibroblast activation may vary and could involve diverse signaling pathways, facilitated by specific molecules also contained within the EVs [56]. For instance, EVs from BC cells can activate fibroblasts and promote their invasive behavior through the transfer of some microRNAs such as microRNA-125-b [57], miR-146a [58], miR-652-5p, miR-126, and miR-185-5p [45] or via survivin [59] and TGF-β/Smad signaling pathway [60]. CAFs often exhibit altered metabolic profiles compared to NFs. EVs can contribute to this reprogramming by delivering metabolic enzymes or signaling molecules that influence the metabolic pathways of fibroblasts. Yan et al. showed that BC-derived EVs containing microRNA-105 induce NFs metabolic reprogramming [61]. In this study, we have demonstrated that TNBC-derived EVs are internalized by NFs, inducing upregulation of CAF markers (MMP1, ITGb, MCT4, FAPa) and pro-inflammatory cytokines (G-CSF, IL8, CXCL10, CXCL12, IL-18, TNFa). This modulation transforms NFs into a more mesenchymal CAF phenotype, enhancing their migration and contractile capabilities.

Investigating the bidirectional communication between BC cells and the TME is a focal point of current cancer research. Disrupting this intricate crosstalk, specifically by interfering with EV-induced fibroblast-to-CAFs transformation, may represent a promising therapeutic approach. This could involve targeting EV biogenesis, release, content, or uptake by blocking surface markers or their receptors. Indeed, EVs often display surface markers that are recognized by receptors on recipient cells [62]. Identifying and targeting these receptors could prevent EV uptake.

On this point, aptamers are promising theragnostic agents for BC because of their unique features, such as high binding affinity, specificity, and low immunogenicity, which make them ideal for diagnostic and therapeutic uses [63]. The ability of aptamers to target specific molecules with few side effects means they may be valid alternatives to antibodies and inhibitors [12, 63, 64].

Here, we have demonstrated by several approaches that the aptamer ex.50.T specifically targets GREM1, a novel EVs diagnostic marker [27] and potential therapeutic target in BC [17, 65]. We employed a pull-down assay using biotinylated ex.50.T followed by mass spectrometry to identify GREM1 as a target protein. We confirmed the binding of ex.50.T to this target protein with the use of recombinant GREM1 protein and several BC cell lines in which the expression of GREM1 was modulated. GREM1 is a soluble, EV-associated protein that is upregulated in various types of tumors; it is involved in multiple biological processes that boost cancer development [22, 66]. We confirmed the presence of GREM1 on BC-specific EVs, validated through immuno-electron microscopy, and its distinctive expression patterns among different BC cell lines and glioblastoma-derived EVs.

Our findings indicate that Ex.50.T binding to GREM1 on BC-EVs hinders the internalization of EVs by NFs, inhibiting their transformation into CAFs. Exposure of NFs to BC EVs increased CAF-associated markers and modified ECM-related proteins, effects inhibited by ex.50.T. Additionally, ex.50.T affected the EVs-induced NFs contractility and significantly reduced the heightened migratory ability induced by BC EVs. Therefore, ex.50.T by inhibiting EVs internalization and GREM1, is directly responsible for counteracting the pro-CAF effects of EVs.

Several molecules have been identified that can block or interfere with EV uptake [67]. For example, in BC, MβCD (methyl-beta-cyclodextrin) has been shown to disrupt caveolin-mediated endocytosis, by depleting cholesterol in the plasma membrane and subsequently reducing the internalization of exosome-sized vesicles [68]. Despite their potential to interfere with EV uptake and functions, they might lack cell-type specificity and have off-target effects [69]. As far as we know, ex.50.T is a novel and innovative tool to specifically disrupt the communication between BC EVs expressing GREM1 and fibroblasts.

Moreover, in BC, GREM1 has been reported to be associated with poor prognosis in patients negative for the ER receptor [17]. GREM1 was found to promote tumor progression [17], and was identified as a mediator of the transition from NFs to CAFs by inhibiting the TGF-β pathway [26]. These findings are epitomized by a phase I/II non-randomized, Open-Label, Multicenter Study using anti-GREM1 antibodies (UCB6114) in patients with advanced solid cancers (NCT04393298) [70]. Therefore, understanding the role of GREM1 in cancer, particularly in modulating CAF behavior and their pro-tumoral properties, is important for elucidating the complexities of tumor-stroma interactions and could potentially offer avenues for targeted therapeutic interventions in cancer. By treating NFs with EVs from BT549 cell lines with stable shRNA-mediated GREM1 knockdown, we observed a reduction in their ability to induce NFs migration and contraction compared to control EVs. On the contrary, treatment of cells with GREM1 recombinant protein fosters NFs migration and contraction. These results establish that GREM1 expression in BT549 EVs plays a pivotal role in activating NF and promoting CAF activation. Our data clearly show that the binding of ex50.T to GREM1 reduced the latter’s ability to induce NFs contraction and migration, a typical feature of the transition into CAFs. Therefore, the aptamer is a promising tool for impairing GREM1’s tumorigenic activity.

Our findings demonstrate that ex.50.T specifically binds to GREM1 and impairs its biological functions. It has more promising effects for clinical use than G-ap49, a GREM1 DNA aptamer [71] developed as a tool to substitute antibodies in the assessment of subcellular localization of the protein. Moreover, ex.50.T exhibits promising potential in inhibiting various oncogenic effects induced by BC EVs on fibroblasts, implicating a prospective role as a therapeutic agent in modulating the TME in BC. However, further comprehensive investigations are warranted to understand its safety profile and its potential for clinical applicability in cancer therapy.

## Materials and methods

### Primary cultures and continuous cell lines

Primary breast cultures were obtained from human surgical biopsy samples provided by Mediterranea Spa and processed as previously reported [14, 45]. Samples were collected according to Helsinki’s declaration, and each subject signed an informed consent before participating in the study. The study was approved by the Research Ethics Committee of the AUO-University of Naples Federico II no. 119/15ES1. Primary breast cultures were grown in Dulbecco’s Modified Eagle’s Medium/Nutrient F12-Ham (DMEM F-12 Sigma-Aldrich, ref. D8437), supplemented with 10% heat-inactivated fetal bovine serum (FBS Sigma-Aldrich, ref. F7524), 1% antibiotic/antimycotic (A/A; Gibco, ref. 15240-062), and 1% amphotericin B (Gibco, ref. 15290-026). Organoids were isolated and cultured according to the protocol reported before [72, 73]. The breast carcinoma continuous cell lines BT-549 and MCF7 were grown in supplemented Roswell Park Memorial Institute Medium (RPMI, Sigma-Aldrich, ref. R8758), while the glioblastoma continuous cell line U87-MG was grown in Dulbecco’s Modified Eagle’s Medium (DMEM, Sigma-Aldrich, ref. D6429). All media were supplemented with 10% FBS and 1% A/A. Human umbilical vein endothelial cells (HUVECs) were grown in M199 Medium (Gibco Life Technologies) supplemented with 20% fetal calf serum (FCS, Gibco Life Technologies), endothelial cell growth factor (100 μg/ml) (Sigma Chemical Co., St. Louis, MO), and porcine heparin (Sigma) (100 μg/ml). HUVECs were used at early passages (I–IV) and grown on plastic surfaces coated with porcine gelatin (Sigma-Aldrich). Cells were routinely tested for mycoplasma contamination using Mycoplasma PCR detection kit (Applied Biological Materials Inc. abm # G238).

### Ex.50.T pull-down assay and mass spectrometry for target identification

Briefly, EVs from primary cell culture of BC patients #37 and #170 and normal fibroadenoma patient #72 were lysed with Triton/r X-100 lysis buffer (Thermo Fisher, ref. J62289-AK) containing 1X protease inhibitor cocktail. The protein lysate (70 µg) was mixed with 500 nM of biotinylated ex.50.T aptamer or a scrambled-biotinylated aptamer for 30 min and subsequently incubated with streptavidin-agarose resin (Thermo Fisher, ref. 20349) for 2 h. The unbound proteins were removed, while the bound proteins were recovered for the proteomic analysis. Before each use, RNA aptamers were subjected to denaturation/renaturation steps at 85 °C for 5 min, 4 °C for 2 min, and 37 °C for 10 min. All incubations were performed at room temperature. To identify the protein targets, LC-MS analysis was implemented as previously reported by our group on three replicates for each experimental point [74, 75].

### Aptamer-based pull-down assay for target validation by Western blot

Upon transfection, U87-MG cells were collected and lysed with a solution of sodium deoxycholate (Sigma-Aldrich, ref. D6750) at 0.1% (w/v) in 10 mM PBS with Ca2^+^, Mg2^+^ (Sigma-Aldrich, ref. D8662). To block non-specific binding sites, the lysates were incubated with 1 ng/µl of yeast RNA (Roche, ref. 10109223001) for 30 min, and then with 50 nM of a biotinylated-scrambled aptamer for 30 min. The lysates were incubated with streptavidin MagneSphere paramagnetic particles (Promega, ref. Z5481) for 30 min. Next, the proteins captured by the particles were removed, while the unbound proteins were incubated with 50 nM of biotinylated ex.50.T aptamer for 30 min, followed by a second incubation with the streptavidin paramagnetic particles. All the incubations were performed at room temperature. The particles were washed 4 times with 200 µl of 10 mM PBS with Ca2^+^, and Mg2^+^, and resuspended in 4x Laemmli Sample Buffer. The samples were denatured for 5 min at 95 °C and analyzed by western blot.

### Aptamer-based ELONA

For the evaluation of ex.50.T binding with GREM1 recombinant protein, microtiter EIA/RIA plates (Corning Costar, ref. 2592) were coated with increasing concentrations of a commercially available GREM1 recombinant protein (R&D System) by overnight incubation at 4 °C. Plates were blocked with 300 μL 3% BSA (Sigma-Aldrich, ref. A3294-100G) in PBS for 2 h, washed once with PBS, and incubated with 200 nM of biotinylated ex.50.T aptamer. After 1 h, plates were washed twice with PBS and incubated with streptavidin-conjugated HRP (Thermo Scientific, ref. N100) dissolved in 100 μL PBS at a 1:5000 dilution. Following three washes, a 3,3,5,5-tetramethylbenzidine substrate solution was added and the colorimetric reaction was stopped with 0.1 M sulfuric acid. The signal intensity was evaluated by measuring the absorbance at 450 nm with a microplate reader (Thermo Fisher Scientific).

### ELISA for GREM1 detection

The amount of GREM1 was determined with ELISA by coating 50 μg of U87MG cell lysate on microtiter EIA/RIA plates (Corning Costar, ref. 2592) by overnight incubation at 4 °C. Plates were blocked with 300 μL of 3% BSA (Sigma-Aldrich, ref. A3294-100G) in PBS for 2 h, washed once with PBS, and incubated with GREM1 antibody (sc-515877) for 1 h at a 1:200 dilution. Plates were then washed twice with PBS and incubated with a mouse HRP antibody dissolved in 100 μL PBS at a 1:2000 dilution. Following three washes, a 3,3,5,5-tetramethylbenzidine substrate solution was added and the colorimetric reaction was stopped with 0.1 M sulfuric acid. The absorbance was measured on a microplate reader at 450 nm.

### Binding assay and quantitative real-time PCR

Binding assays on cells were performed by seeding 2 × 10^5^ cells in 6-well plates. Cells were pre-treated with 0.2 µg/µl of baker’s yeast tRNA (Roche, ref. 10109495001) to block non-specific sites, and then incubated with 200 nM of ex.50.T aptamer. Both incubations were performed at 37 °C for 30 min in a serum-free medium. Following three washes with serum-free medium, the bound RNAs were recovered by TRIzol containing 0.5 pmol/mL of an unrelated aptamer (CL4: 5′-GCCUUAGUAACGUGCUUUGAUGUCGAUUCGACAGGAGGC-3′) used as a reference control. The amount of bound aptamers was determined by qRT-PCR with iTaq Universal SYBR green Supermix (BioRad), and calculated in relation to a standard curve of known RNAs and normalized to the CL4 reference control and to cell number. For GREM1 mRNA quantification, the following primers were used: β-Actin FW-5’- TGC GTG ACA TTA AGG AGA AG-3’, β-Actin RV- 5’-GCT CGT AGC TCT TCT CCA-3’; and GREM1 FW 5’-CAT GTG ACG GAG CGC AAA TA-3’, Gremlin-1 RV 5’-GTT CAG GGC AGT TGA GTG TG-3’). RNA was retrotranscribed with Superscript III First Strand cDNA synthesis kit (Thermo Fisher Scientific) and qRT-PCR was performed with iTaq Universal SYBR green Supermix (BioRad) at an annealing temperature of 60 °C.

### Binding and internalization assay by flow cytometry-analysis

Binding assay on cells was performed by seeding 2 × 10^5^ cells in 6-well plates. Cells were pre-treated with 0.2 µg/µl of baker’s yeast tRNA to block non-specific sites and then incubated with 200 nM of ex.50.T-Alexa680 aptamer. Both incubations were performed at 37 °C for 30 min in a serum-free medium. Following three washes with serum-free medium, the bound RNA was measured as the intensity of fluorescence of Alexa 680 with a BD Accuri C6 Flow cytometer (Thermo Fisher Scientific). For internalization assay, after the binding with Alexa 680-ex.50.T aptamer, cells were incubated with a solution of 0.5 M of NaCl in PBS for 5 min at 4 °C to remove aptamers exposed on the cellular surface and then analyzed by flow cytometry.

### SPR experiments

SPR experiments were carried out on a Biacore X100 (GE Healthcare, Uppsala, Sweden). Grem1 protein was immobilized on a research-grade CM5 sensor chip using the amine-coupling chemistry and the HBS-EP as running buffer (10 mM HEPES, 150 mM NaCl, 3 mM EDTA, 0.005% surfactant P20, pH 7.4) as described elsewhere [76, 77]. The protein (50 μg/mL in 10 mM sodium acetate, pH 5) was immobilized on the sample flow cell, leaving the reference cell as blank. Single-Cycle Kinetics (SCK) experiments were performed at 25 °C by injecting increasing concentrations of RNA samples (from 0.062 to 1 μM) at a flow rate of 30 μL/min. For these experiments, a running buffer consisting of PBS (137 mM NaCl, 2.7 mM KCl, 10 mM NaH2PO4/Na2HPO4, 1.8 mM KH2PO4/K2HPO4, pH = 7.4) was used, and association and dissociation times were set at 60 and 600 s, respectively. Curves obtained on the reference surface were subtracted from those recorded on the protein-functionalized surface, to eliminate the effects of refractive index changes due to the buffer. Data were fitted to a 1:1 kinetic interaction model, using the global data analysis option available within the BiaEvaluation software (GE Healthcare, Uppsala, Sweden) provided with the instrument.

### In silico GREM1 protein preparation

The crystallographic structure of the GREM1 (residues 71–184, chain A) was retrieved from the Protein Data Bank (PDB code: 5AEJ) [78] and employed for molecular docking and molecular dynamics (MD) simulations with the RNA aptamer ex.50.T. The structure was prepared by employing the graphical interface of Schrödinger’s molecular modeling platform, Maestro v.12.7.156. In detail, the protein was prepared with the Protein Preparation Wizard [79] included in Maestro. Missing hydrogen atoms were added, and crystallographic water molecules were deleted. The N-terminal and C-terminal residues were capped with the acetyl (ACE) and N-methyl amide (NME) groups, respectively. The PROPKA [80] program, included in Maestro, was employed to properly describe the protonation state of the protein residues and the hydrogen bonds network at neutral pH. Finally, a relaxation procedure was performed by running a restrained minimization only on hydrogen atoms using the OPLS2005 [81] force field.

### In silico aptamer conformational prediction

The ex.50.T aptamer 1D sequence (5’-uGuGGcAGuuAAGAAuAGAucuucGcuGcGAuu-3’) was used as input for bioinformatics tools relying on different models to predict the 2D structure. In particular, we evaluated the predictions of 5 different programs, namely RNAfold^46^, RNAstructure [31], and Mfold [32] employing a so-called thermodynamic model (or Turner’s nearest-neighbor model) [30], pKiss webserver [33], which allows predicting RNA structures with kissing hairpin motifs in an arbitrarily nested fashion, and finally MXfold2 [34], which is based on a deep-learning approach integrating folding scores calculated by a deep neural network with a thermodynamic model. The five software converged on the same solution, prompting us to employ the predicted 2D structure as input for the subsequent 3D model. For this step, we used the RNAComposer software [35]. RNAComposer first assigns the appropriate 3D structure based on the knowledge of all 3D RNA structures deposited into the RCSB PDB (RNA FRABASE database) [82], and then minimizes the resultant structure in the torsion angle space and the Cartesian atom coordinate space to produce the final output. This program has already demonstrated in several cases to provide highly accurate predictions in terms of base pairing (canonical and noncanonical) and stacking [36–38].

### Molecular docking and molecular dynamics simulations

The 3D RNA aptamer conformation described above was employed for *consensus* docking against the prepared GREM1 crystallographic structure. This strategy relied on 5 different programs, encompassing rigid-body and semi-flexible molecular algorithms. For rigid-body docking, we used ZDOCK [39], HDOCK [40], PATCHDock [41], and NPDock [42], while HADDOCK [43] was employed as a semi-flexible docking program. For each program, the 10 best-scored lowest-in-energy complexes were selected and underwent a cluster analysis using the Gromos algorithm implemented in Gromacs [83] to check the convergence of the predictions. The centroid structure of the most abundant cluster (RMSD cutoff of 8 Å) was employed for the subsequent MD simulation. A 1.5 μs MD simulation was performed by using the GROMACS software [83]. The AMBER ff14SB [84] force field was employed for the protein, while the χOL3 force field [85] was used for the RNA aptamer. The leap program available in AmberTools was used to prepare the complex for the molecular dynamics simulations. The system was solvated in a cubic water box where the minimum distance between any solute atom and the edge of the box was 12 Å. The TIP3P model [86] was used to treat added water. The system’s neutrality was ensured by adding monovalent sodium ions, modeled with Joung and Cheatham [87] parameters. Finally, coordinates and topology files for the whole system were obtained. For both the equilibration and production runs, the Verlet cut-off scheme was used for non-bonded interactions neighbor search, the smooth Particle-Mesh Ewald (SPME) method [88] was employed for long-range electrostatic interactions, while the cut-off for Van der Waals interactions was set to 1.2 nm. The equilibration procedure consisted of two energy minimization steps followed by subsequent NVT and NPT runs. The energy minimization consisted of two stages performed by using the steepest descent algorithm: (1) 20000 steps with harmonic restraints of 1000 kJ mol^−1^ nm^−2^ applied to the RNA and the protein heavy atoms, so that only the solvent was unconstrained; (2) 20000 steps during which the entire system was allowed to relax. Only for these runs, a gradual decrease in the Coulomb and Lennard-Jones potentials was imposed between 1 and 1.2 nm. After energy minimization, for the MD simulations (either equilibration or production), the leap-frog [89] algorithm for integrating Newton’s equations of motion was used and a time step of 2 fs was chosen; accordingly, the LINCS [90] algorithm was employed to constrain bonds involving hydrogen atoms. During the MD equilibration procedure, the system was gradually heated by increasing the temperature with subsequent MD runs in the canonical ensemble (NVT) using the weak-coupling Berendsen [91] scheme. In detail, three 500 ps NVT steps were performed by gradually increasing the temperature of 100 K up to 300 K. At each step, harmonic restraints were applied to all the heavy atoms of both the protein and the aptamer and were gradually decreased from 1000, 500 to 250 kJ mol^−1^ nm^−2^. Then, two NPT equilibration runs of 1 and 5 ns, respectively, were performed to adjust the box volume, using the Berendsen algorithm for pressure coupling. In the first NPT step restraints of 50 kJ mol^−1^ nm^−2^ were applied on heavy atoms, while no restraints were used in the last equilibration run. For the 1.5 μs production run, temperature and pressure controls were carried out with the velocity-rescale [92] and Parrinello-Rhaman [93] schemes, respectively. The trajectory visualization and the RMSD analyses were performed with the VMD software. The presented figures were obtained using Pymol (https://www.schrodinger.com/products/pymol), Chimera*X* [94], and the image manipulation program Gimp (2.10.22 revision 3) (https://www.gimp.org), while the graphics were made with the Xmgrace software (Paul J. Turner Center for Coastal and Land-Margin Research Oregon Graduate Institute of Science and Technology Beaverton, Oregon).

### Western blotting

Proteins were analyzed by Western blot as reported previously [95–97]. Primary antibodies were as follows (from Santa Cruz Biotechnology, unless otherwise stated): anti-GREM1 (sc-515877; AF956, R&D System; and 4383, Cell Signaling Technology); anti-PDGFR (3164, Cell Signaling Technology); anti-β actin (A5441, Sigma- Aldrich); anti-pVEGFR2 (MA5-15170; ThermoFisher); anti-VEGFR2 (9698; Cell Signaling Technology); anti-CD9 (sc-13118); anti-CD81 (sc-166029); anti-TSG101 (sc-6037); anti-ALIX, (sc-53540); anti-MMP1 (sc-21731); anti-ITGβ1 (sc-374429); anti-MCT4 (sc-376140); and anti-GPDH (sc-365062). HRP-conjugated secondary antibodies were from Santa Cruz Biotechnology.

Proteome Profiler Antibody Array-Human Cytokine (R&D System) were performed according to manufacture instructions with 500 microliter of conditioned medium of NF#103 treated with BT-549 EVs alone or in combination with Ctrl-Apt or ex.50.T.

### Modulation of gremlin-1 expression in breast cancer cells

Lentiviral-mediated overexpression was obtained with Lenti ORF clone of Human gremlin 1 (GREMLIN1), transcript variant 1, mGFP tagged from Origene (CAT#: RC210835L2). The GREMLIN1 knockdown was obtained with the Human shRNA Gremlin-1 Kit (Locus ID 26585) from Origene (CAT# TL312620). Plasmids were cotransfected with Lenti-vpak packaging kit (Origene) into 293FT cells using the provided transfection kit (Origene). Knockdown and overexpression were confirmed using Western blot and qPCR. For transient gremlin-1 down-modulation, the human gremlin-1 siRNA (sc-39408, Santa Cruz Biotechnology) was transfected using lipo2000 (Thermo Fisher Scientific) at 200 nMol.

### EV isolation

EVs were isolated from cell culture media of BT-549, MCF7, and U87MG cells. In detail, 3 × 10^6^ cells were plated in 150 mm cell culture dishes in 12 ml of RPMI medium (Sigma-Aldrich) supplemented with 10% Exo-FBS (FBS depleted of exosomes, SBI, System Biosciences), 1X antibiotic-antimycotics. After 72 h, culture media were collected and centrifuged at 3000 × *g* for 15 min at room temperature (RT) to remove cellular debris. The supernatant was transferred to ultracentrifuge sterile tubes (Beckman coulter centrifuge tubes) and centrifuged at 4 °C at 45,000 rpm. The supernatant was carefully removed, and EVs-containing pellets were resuspended in 5 mL of ice-cold PBS and a repeated 45,000 rpm centrifugation at 4 °C was carried out. The supernatant was discarded and the EVs pellet was resuspended in 200 μL of PBS and stored in −80 °C for future use.

### Transmission electron microscopy (TEM)

For examination of immuno-gold-labeled EVs isolated from BT459, MCF7, and U87MG cell cultures, the mouse monoclonal antibody sc-515877 directed against gremlin-1 (Santa Cruz Biotechnology, Santa Cruz, CA) was used; the secondary antibody used was a Protein A gold-conjugate (10 nm) to revealed antigen staining. Five µl (10 × 10^5^–14 × 10^5^/ml) of unfixed purified EVs were diluted in 5 µl of fixative containing 2% paraformaldehyde for 5 min to dilute the concentration to 1/2. The sample was loaded on a formvar film coated 100 mesh copper EM grid and incubated for 10 min at room temperature for adhesion. The excess sample on the grid was removed by contacting the grid edge with filter paper. Next, the grid was placed 3 times on drops of 0.2% glycine/PBS 1X for 2 min and then transferred to a drop of blocking with 1% bovine serum albumin (BSA) for 20 min. This was followed by an incubation step on the drop of the primary antibody dilution (1:30 dilution in 1% BSA/PBS 1X) for 2 h at room temperature. Afterward, grids were washed 2 times for 5 min in drops of 0.1% BSA/PBS 1X and binding of antibodies was detected with an additional incubation with Protein A conjugated with 10 nm colloidal gold (PAG, purchased from Cell Microscopy Core Department of Cell Biology University Medical Center Utrecht, Utrecht, The Netherlands) (dilution 1:100 in 1% BSA/PBS 1X) was performed for 30 min at room temperature. Grids were then rewashed 3 times for 5 min in filtered PBS 1X followed by post-fixation with 1% glutaraldehyde, stained using 1.5% UA solution in water for 10 min. The excess UA solution on the grid was removed by contacting the grid edge with filter paper. Grids were washed 6 times for 1 min on a drop of filtered distilled water to remove the excess staining solution and air-dried overnight. Images were acquired from grids using a FEI Tecnai 12 120 kV transmission electron microscope (FEI Company, The Netherlands) equipped with a Veleta CCD digital camera (Olympus Soft Imaging Solutions GmbH, Münster, Germany) and operating at 120 kV. Images were collected at magnifications of ×120,000.

For Ex.50.T labeling, gold nanoparticles (AuNPs) with a diameter of 20 ± 3 nm were functionalized with aptamers using an optimized freezing method. UV-Vis spectroscopy confirmed the functionalization’s success, where a red-shift in the resonance peak indicated successful aptamer attachment to the AuNPs. The morphology of both AuNPs and AuNPs bound to EVs (fAuNPs) was characterized using transmission electron microscopy (TEM) with a JEOL JEM-2100 microscope (JEOL Ltd., Japan). To avoid overcrowded micrograph images, the exosome samples were diluted 1000-fold in MilliQ water, while the gold nanoparticles were at OD 1. Before deposition on the TEM grid, the AuNP/exosome mixture was prepared using a microfluidic system optimized to enhance binding efficiency.

### PKH 26 exosome labeling and immunofluorescence

EVs (50 μg) were stained for 5 min in the dark at room temperature with PKH26 Red Fluorescent Cell Linker (0.33 μl; Sigma-Aldrich) in a final reaction volume of 2 ml. Then an equal volume of PBS 1% BSA was added to block the labeling reaction. To pellet the EVs, ultracentrifuge for 70 min at 31,000 rpm at 4 °C was performed. The PKH labeled-EVs were washed with 5 ml PBS and centrifuged for 70 min at 31,000 rpm and the pellet was resuspended in PBS (100 μL). For EVs uptake analysis, EVs were incubated with ex.50.T aptamer, or control aptamer (200 nM), in 300 μL of DMEM/F12-10% FBS exosome-free media for 30 min in the dark at room temperature with gentle rotation. The mix of EVs, aptamers, and media was used to treat the fibroblasts.

For immunofluorescence analysis, the fibroblasts were fixed with an ice-cold mix of methanol/acetone (1:1) for 10 min at −20 °C. Subsequently, cells were washed twice with PBS and then blocked in PBS/1% BSA for 30 min at room temperature, and, after two washes with PBS, incubated with anti-FAPα (1:50), anti-MCT4 (1:50), anti-ITG-β1 (1:50) and, anti-β-actin (1:1000) primary antibody, diluted in blocking solution for ON at 4 °C. After two washes with PBS, FITC-anti-mouse secondary antibodies (Santa Cruz Biotechnology) were added for 1 h at 37 °C (1:200). Cells were then incubated with DAPI (1:1000) (BD Pharmingen) for 10 min at RT in dark for nuclei visualization. Coverslips were washed twice with PBS and once with water, mounted with 3 μl of 1:1 glycerol (Sigma-Aldrich) in PBS on a microscope slide and the cells were visualized by confocal microscopy (LSM 700, Zeiss, Milan, Italy).

### Wound healing assay

Primary normal fibroblast cells (2 × 10^5^) from different non-cancerous biopsies were seeded in 24 well-plates in DMEM F12 supplemented with 10% exosome-free FBS. Following 24 h, a wound was scraped with a 20-μL tip in each well, cells were then washed with PBS and treated with 50 μg of BT-549-derived EVs pre-incubated with and without ex.50.T aptamer or CtrlApt (500 nM) in 300 μL of serum-free DMEM F12 medium. Cells were incubated for 24 h at 37 °C in humidified 5% CO_2_. Pictures were taken soon after the wound creation (T0) and 24 h after the wound creation (T24). Scratched areas were manually traced and measured with ImageJ Software.

### Migration assay

Normal primary fibroblasts were seeded at 1 × 10^5^ cells into the upper chamber of a 24-well transwell (Corning Incorporated) in DMEM/F12 supplemented with 1% exosome-free FBS containing 50 μg BT-549-derived EVs pre-incubated with ex.50.T aptamer or with CtrlApt (500 nM). The lower chamber of the transwell was filled with 600 μl of media complemented with 10% FBS, used as a chemoattractant. After 24 h of incubation at 37 °C in humidified 5% CO_2_, migrated cells were visualized by staining with 0.1% crystal Violet in 25% methanol. Non-migrated cells were scraped off the top of the transwell with a cotton swab. The percentage of migrated cells was evaluated by eluting crystal violet with 1% SDS and reading the absorbance at *λ* 590 nm.

### Collagen contraction assay

Collagen contraction assay was carried out using 1.25 × 10^4^ NFs in 24-well plates (Corning) as previously described [45]. A reaction mix composed of rat tail type I collagen (Corning, Milan, Italy), MEM 10X (Sigma-Aldrich, Milan Italy), acetic acid 5 mM, and NaOH 1 M was prepared and added to the fibroblasts (5 × 10^4^) resuspended in FBS-exosome free. 1.3 ml of that mixture was plated into a 12-well plate and incubated at 37 °C until polymerization. After 4 h the pugs were detached from the edges of the wells by using a syringe and then NFs were treated with 10% FBS-exosome-free, DMEM F/12 containing 50 μg BT-549-derived EVs pre-incubated with ex.50.T aptamer or with CtrlApt (500 nM) or with recombinant GREM1- 6xhistag (expressed in HEK293T cells and purified as previously described [19]) and with 500 nMol of ex.50.T. After 14 h of incubation at 37 °C in humidified 5% CO_2,_ the images were taken using a mobile phone’s camera app held steady in a fixed location and the plug areas were computed using ImageJ software.

### Statistical analysis

Continuous variables are reported as mean ± standard deviation. Statistical significance was set at the two-tailed 0.05 level. Computations were performed with Prism GraphPad version 6.00 for Windows, GraphPad Software, Boston, Massachusetts USA, www.graphpad.com. Statistical significance (*p* < 0.05, *p* < 0.01, *p* < 0.001) was assessed using Student’s *t* test (for comparisons between two groups), and one-way or two-way ANOVA coupled with Tukey’s post hoc testing (for multiple comparisons).

## Supplementary information

Supplementary information

uncropped films Wb

## Data Availability

The data generated in this study are available upon request from the corresponding author. Uncropped western blot images are uploaded as Supplementary Information. Full and uncropped western blot are loaded as Supplementary Information.
