# Supplementary material for: Ex.50.T aptamer impairs tumor–stroma cross-talk in breast cancer by targeting gremlin-1
Source: Cell Death Discov. 2025 Mar 11;11:94. doi: 10.1038/s41420-025-02363-6 (PMC11897156; doi:10.1038/s41420-025-02363-6)
Supplement: Supplementary file 1 — Supplementary information [file 41420_2025_2363_MOESM1_ESM.docx]

**Supporting information**

**Ex.50.T aptamer impairs tumor–stroma cross-talk in breast cancer by targeting gremlin-1**

Cristina Quintavalle^1§*^, Francesco Ingenito^2§^, Giuseppina Roscigno^2§^, Birlipta Pattanayak^2^, Carla Lucia Esposito^1^, Alessandra Affinito^2,3^, Danilo Fiore^1,2^, Gianluca Petrillo^2^, Silvia Nuzzo^4^, Bartolomeo Della Ventura^5^, Federica D’Aria^6^, Concetta Giancola^6^, Stefania Mitola^7^, Elisabetta Grillo^7^, Marinella Pirozzi^1^, Greta Donati^6^ , Francesco Saverio Di Leva^6^, Luciana Marinelli^6^, Zoran Minic^8^, Francesca De Micco^9^, Guglielmo Thomas^9^, Maxim V. Berezovski^8^, and, Gerolama Condorelli^1,2,*^

*^1^ Institute of Endotypes in Oncology, Metabolism and Immunology "G. Salvatore* (IEOMI), Consiglio Nazionale delle Ricerche (CNR), Naples, Italy

*^2^*Department of Molecular Medicine and Medical Biotechnology, “Federico II” University of Naples

*^3^* AKA Biotech S.r.l., Napoli, Italy

*^4^* IRCCS SYNLAB SDN, Naples, Italy

*^5^*Department of Physics "Ettore Pancini", University of Naples "Federico II", Naples, Italy.

*^6^*Department of Pharmacy, University of Naples Federico II, 80131 Naples, Italy.

*^7^*Department of Molecular and Translational Medicine, University of Brescia, Brescia, Italy

*^8^*Department of Chemistry and Biomolecular Sciences and John L. Holmes Mass Spectrometry Facility, University of Ottawa, Ottawa, Ontario K1N 6N5, Canada

*^9^*Mediterranea Cardiocentro, Napoli, Italy

*^§^*Co-first author

*Correspondence should be addressed to Gerolama Condorelli Department of Molecular Medicine and Medical Biotechnology, “Federico II” University of Naples, V. Tommaso de Amicis 95, 80131 Naples, Italy, tel. +390815452921, fax. +390817462685, Email [gecondor@unina.it](mailto:gecondor@unina.it); Cristina Quintavalle IEOMI, CNR, Naples, Italy. Via Pansini 5, 80131 Naples, Italy, email: [cristina.quintavalle@ieos.cnr.it](mailto:cristina.quintavalle@ieos.cnr.it), tel. +390815452921, fax. +39 0812296674.

**Supplementary Tables**

| **Patient** | **Total elements** | **ex.50.T Elements** |
| --- | --- | --- |
| #72 | **51** | PIP,FLG2,HSPD1,HSP90AB1,ANXA2,VCP,KRT19,RPL22,ENO1,HSP90B1,FLNA,CALML3,NCL,HSPA5,DEFA1,DEFA1B,TUBB,PRSS2,HNRNPA1,LCN1,COL6A3,HNRNPA2B1,LMNA,PPIA,HSPA8,CASP14,COL1A1,ANXA1,TUBB4B,MYH9,DSG1,GAPDH,CALML5,JUP,IGKC,EEF1A1,PFN1,TYMP;CO2,ATP5A1,KRT13,YWHAG,AHNAK,RPS27A,KRT17,HSP90AA1,P4HB,SBSN,S100A9,PKM,YWHAZ,YWHAE,HRNR |
| #37 | **21** | FLG2,SFRP4,ATP5A1,PRSS2,GREM1,DSP,RPS27A,CLEC3B,FN1,CCDC80,THBS1,KRT17,COL1A1,POSTN,DSG1,IGFBP7,LYZ,CSN2,CALML5,CTGF,HRNR |
| #170 | **9** | KRT6B, KRT16, KRT10,TGFBI,KRT6A,PRSS2,GREM1,DSP,CCDC80 |

**Supplementary Table 1.** Venn Diagram analysis of proteins identified in ex50.T pulldown assay in patient #72, #170, and #72 (q-value<0.001).

| **Patient** | **Total elements** | **Elements** |
| --- | --- | --- |
| #170, #37, # 72 | 1 | PRSS2 |
| #170, #37 | 3 | CCDC80, GREM1, DSP |
| #37, #72 | 8 | FLG2,COL1A1,DSG1,CALML5,ATP5A1,RPS27A,KRT17, HRNR |
| #37 | 9 | SFRP4,CLEC3B,POSTN,CTGF,FN1,THBS1,IGFBP7,LYZ, CSN2 |
| #170 | 5 | KRT6B,KRT6A,KRT10,KRT16,TGFBI |
| #72 | 42 | PIP,HSPD1,HSP90AB1,ANXA2,VCP,KRT19,ENO1,RPL22, CALML3,FLNA,HSP90B1,NCL,HSPA5,TUBB,DEFA1, DEFA1B,HNRNPA1,LCN1,COL6A3,PPIA,LMNA,HNRNPA2B1,HSPA8,CASP14,ANXA1,MYH9,TUBB4B,GAPDH,JUP, IGKC,EEF1A1,PFN1,TYMP,SCO2,KRT13,YWHAG,AHNAK,HSP90AA1,P4HB,SBSN,S100A9,PKM,YWHAZ,YWHAE |

**Supplementary Table 2.**  Venn diagram analysis of proteins identified in ex50.T pulldown assay in patients #170 and #37 but not in #72 (q-value<0.001).

**Supplementary Figures**


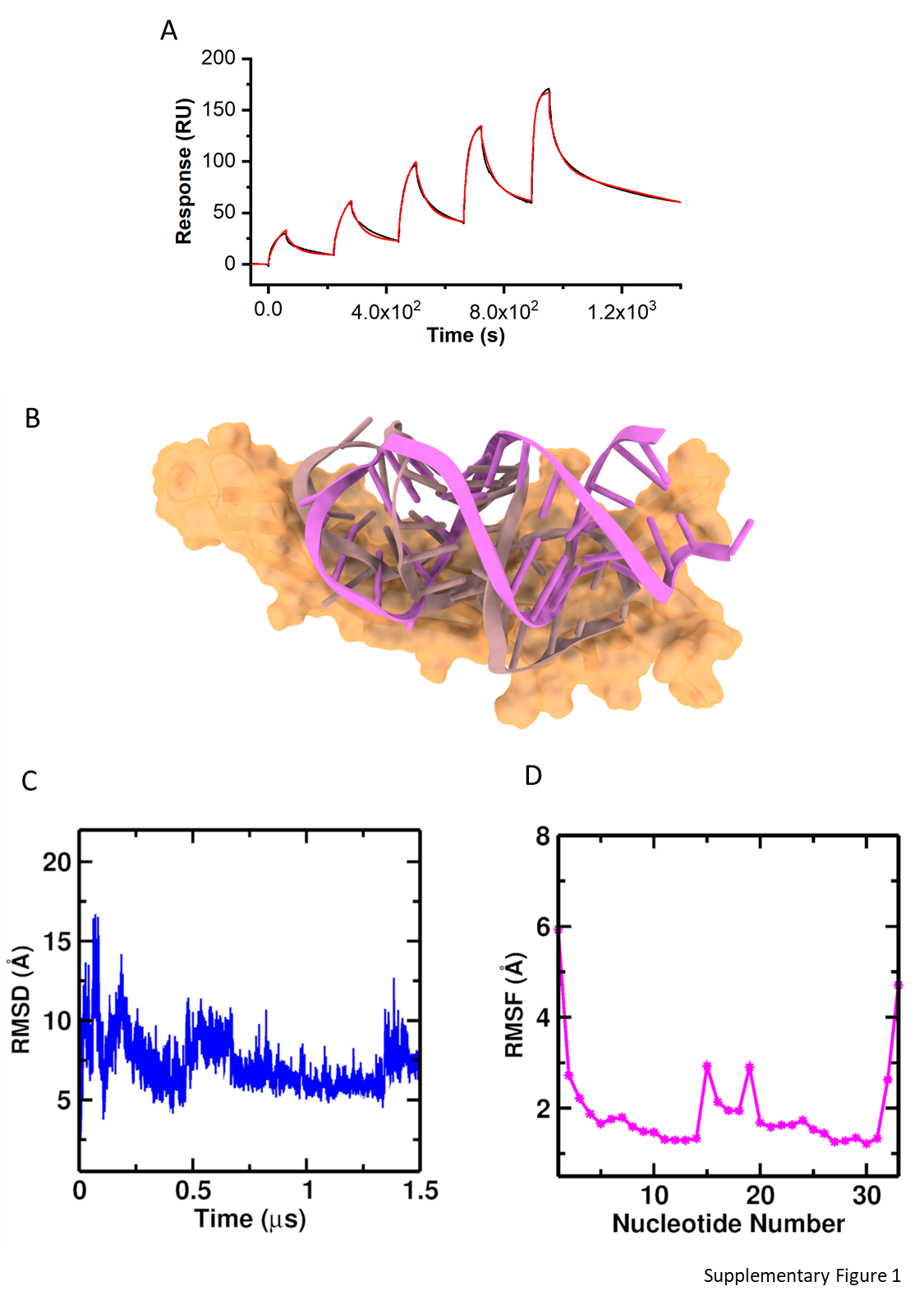


**Supplementary Figure 1- (A)** Time evolution SPR sensorgrams obtained at 25 °C by injection of increasing concentrations of Ex.50.T aptamer (from 0.062 to 1 μM) on the chip-immobilized GREM1 protein. **(B)** Schematic representation of the ex.50.T-GREM1 complex from *consensus* docking overlapped with the aptamer conformation predicted by MD simulation. GREM1 is shown as an orange surface, the aptamer as ribbon and stub (light brown for the docking pose and magenta for the MD conformation). **(C)** Ex.50.T heavy atoms RMSD during the MD simulation. Prior to RMSD calculation, the trajectory was aligned on the Cα of the protein secondary structural elements with respect to the initial MD frame. **(D)** Ex.50.T nucleotides RMSF during the MD simulation. The analysis was performed on the aptamer heavy atoms.


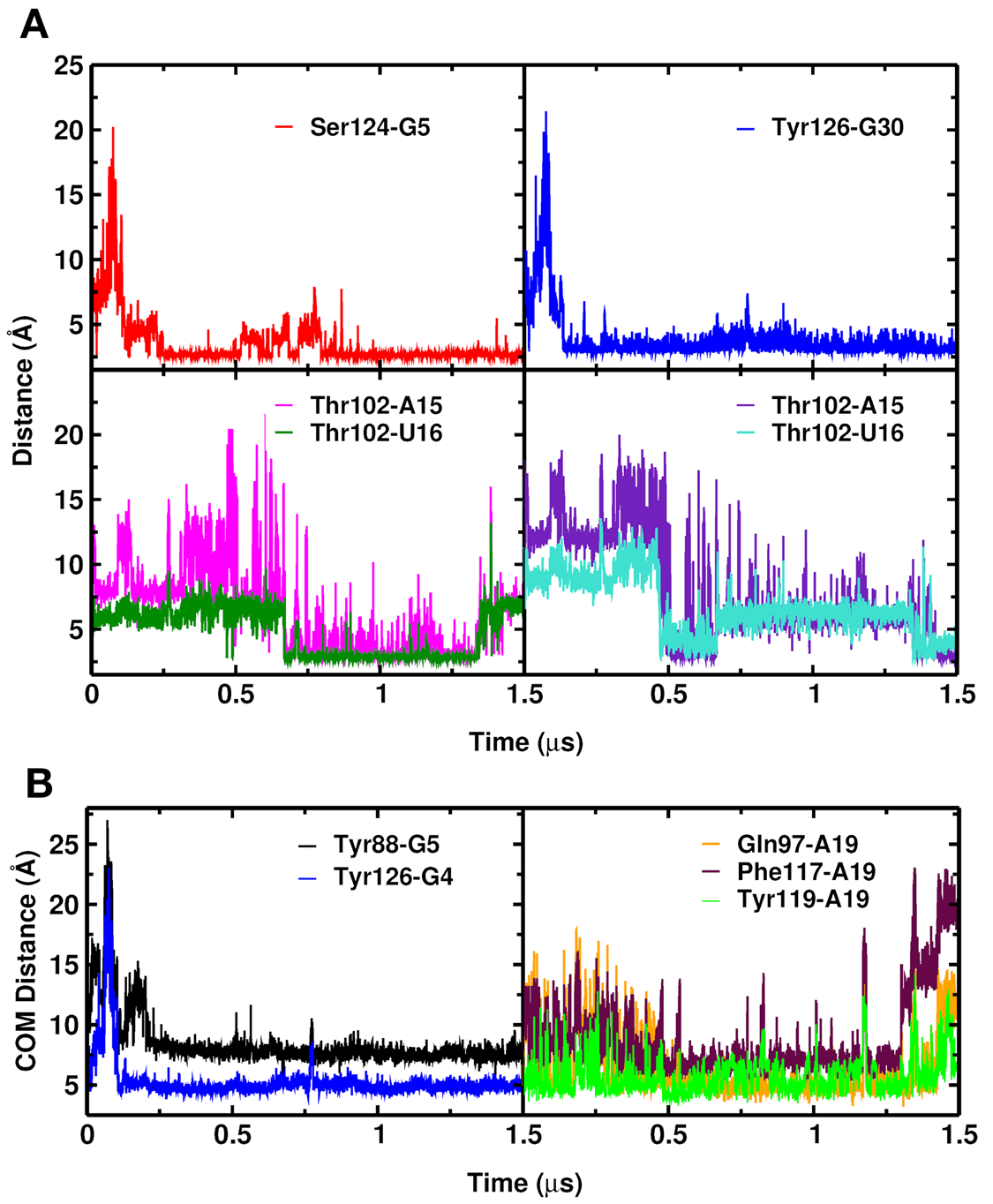


**Supplementary Figure 2- (A)** Time evolution of the distance between: Ser124 hydroxyl oxygen and G5 backbone phosphate oxygen (top left); Tyr126 phenolic oxygen and G30 amine group (top right); Thr102 backbone amide oxygen and A15 amine nitrogen, and Thr102 backbone amide nitrogen and U16 pyrimidine oxygen (bottom left); Thr102 hydroxyl oxygen and either A15 amine nitrogen or U16 pyrimidine nitrogen (bottom right). **(B)** Time evolution of distance between the center of mass (heavy atoms only) of the following pairs: Tyr88 (phenolic ring)-G5 (pyrimidine ring) and Tyr126 (phenolic ring)-G4 (pyrimidine ring) (left panel); Gln97 (amide group)-A19 (pyrimidine ring), Phe117 (phenyl ring)-A19 (pyrimidine ring), Tyr119 (phenolic ring)-A19 (imidazole ring) (right panel).

**
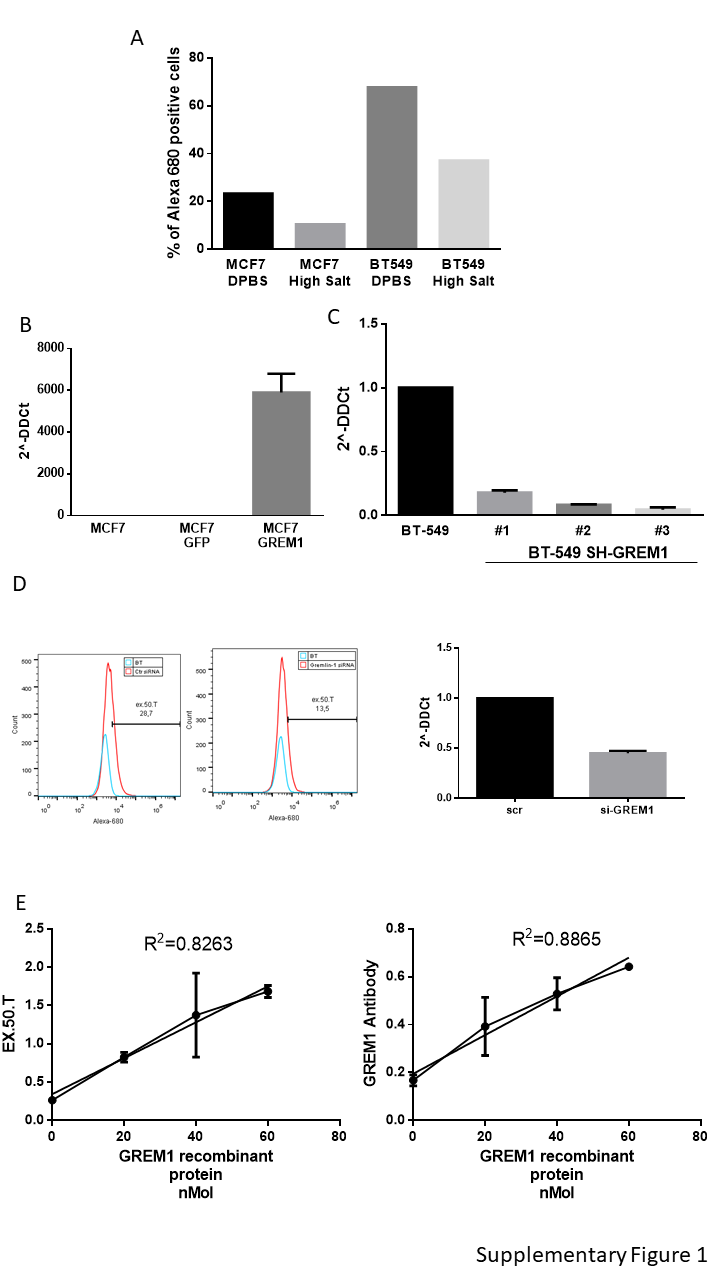
**

**Supplementary Figure 3- (A)** Percentage of bound and internalized Alexa-680 ex.50.T aptamer in MCF7 and BT549 cell lines. **(B,C)** qPCR of mRNA gremli-1 in MCF7 cells overexpressing gremlin-1 and BT549 interfered for gremlin-1 expression, respectively. **(D)** Cytofluorometric analysis of Cy3-ex.50T in BT549 transiently transfected with gremlin-1 siRNA and an shRNA control (left). qPCR analysis of shRNA effect on gremlin-1 mRNA expression (right). **(E)** ELONA and ELISA on recombinant gremlin-1 protein at different doses as indicated. Linear regression was used to measure the correlation (R^2^) between ex.50.T and gremlin-1 antibody and absorbance.

**
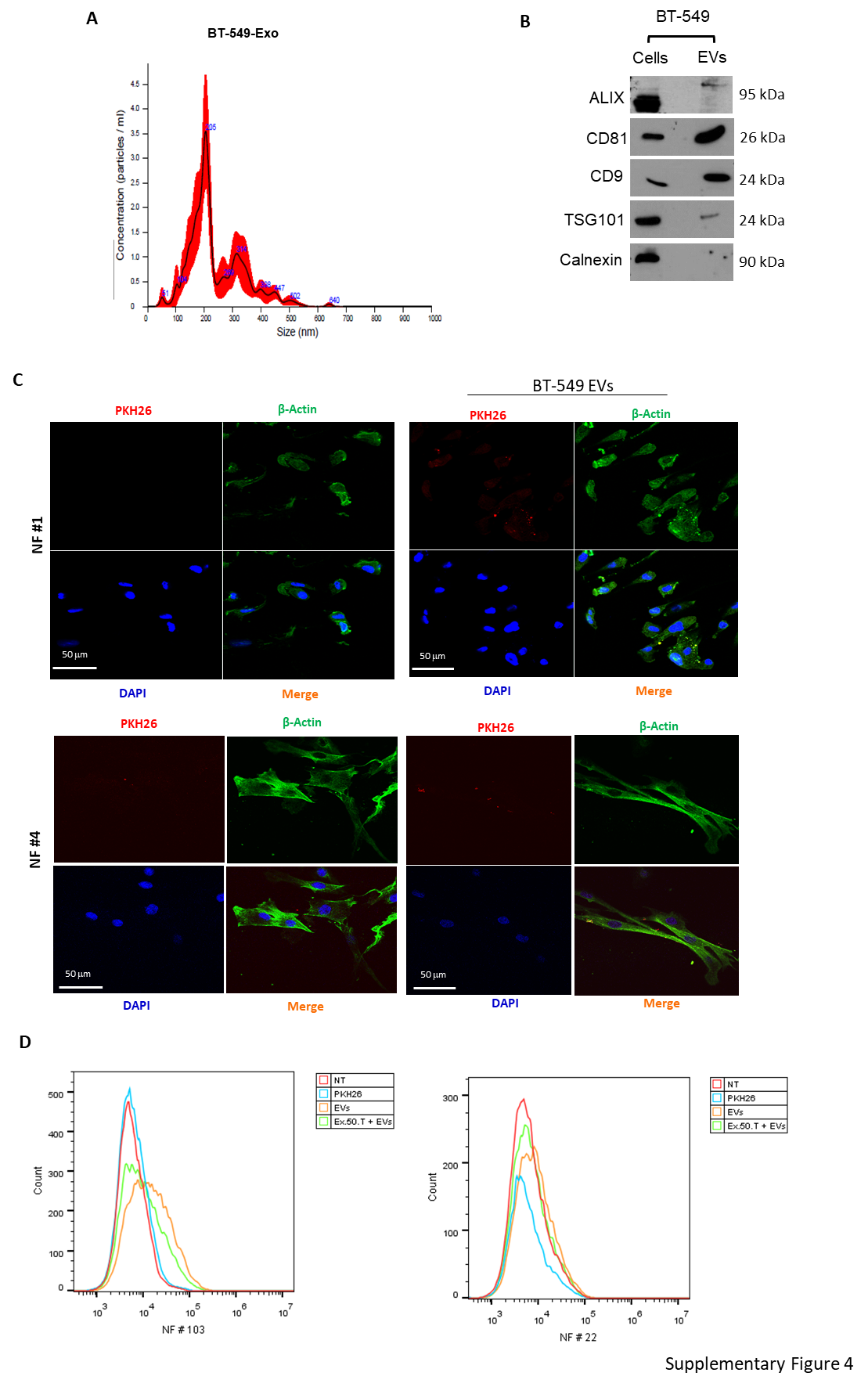
**

**Supplementary Figure 4-** **(A)** Study of distribution and size with NTA of BT549-derived EVs: the average diameter of the vesicles is in the range of 20–250 nm. **(B)** Vesicles secreted by BT549 cells were assessed by western blot for the positivity to EV markers (CD63, CD9, CD-81, TSG101, Alix) and for the lack of calnexin, an endoplasmic reticulum marker that confirms non-contamination of EVs with cellular components. **(C)** Representative images from confocal microscopy of normal fibroblasts (NFs) from two patients, exposed to PKH26-labeled, BT-549-derived EVs. The image shows reduced EV internalization, as evinced by the decreased merged signal (orange). PKH-26 alone was used as negative control (NT). All images were captured at the same settings, enabling direct comparison of staining patterns. NFs were stained using DAPI (blue) and ALEXA488-conjugated anti-β-actin antibody (green), respectively for nuclei and cytoskeleton detection. Magnification 63x. **(D)** NFs were seeded and incubated with PKH-26-stained BT549-derived EVs or PKH alone. After 3h, cells were prepared for flow cytometry. Histogram of percentage internalization, showing an increase of EVs uptake. PKH-26 without EVs was used as negative control. The experiment was performed once.


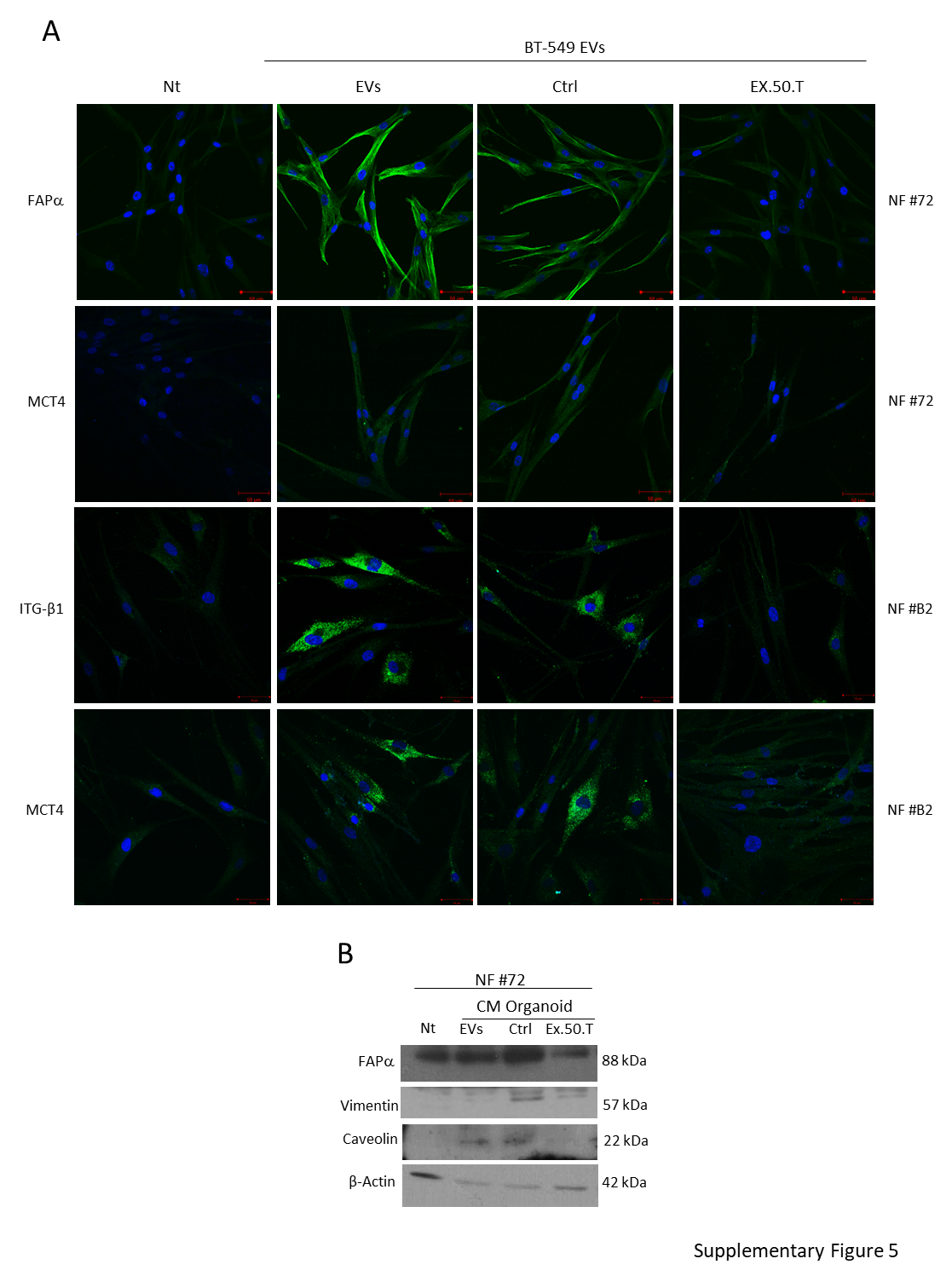


**Supplementary Figure 5-** **(A)** Representative Western blot of CAF markers associated with NF activation into CAFs upon exposure to conditioned medium from BC organoid #213, CtrlApt, and ex.50.T. Beta-actin and GAPDH were used as loading control. **(B)** Representative Western blot of CAF markers associated with NF activation into CAFs upon exposure to conditioned medium from BC organoid #213, CtrlApt, and ex.50.T. Beta-actin and GAPDH were used as loading control


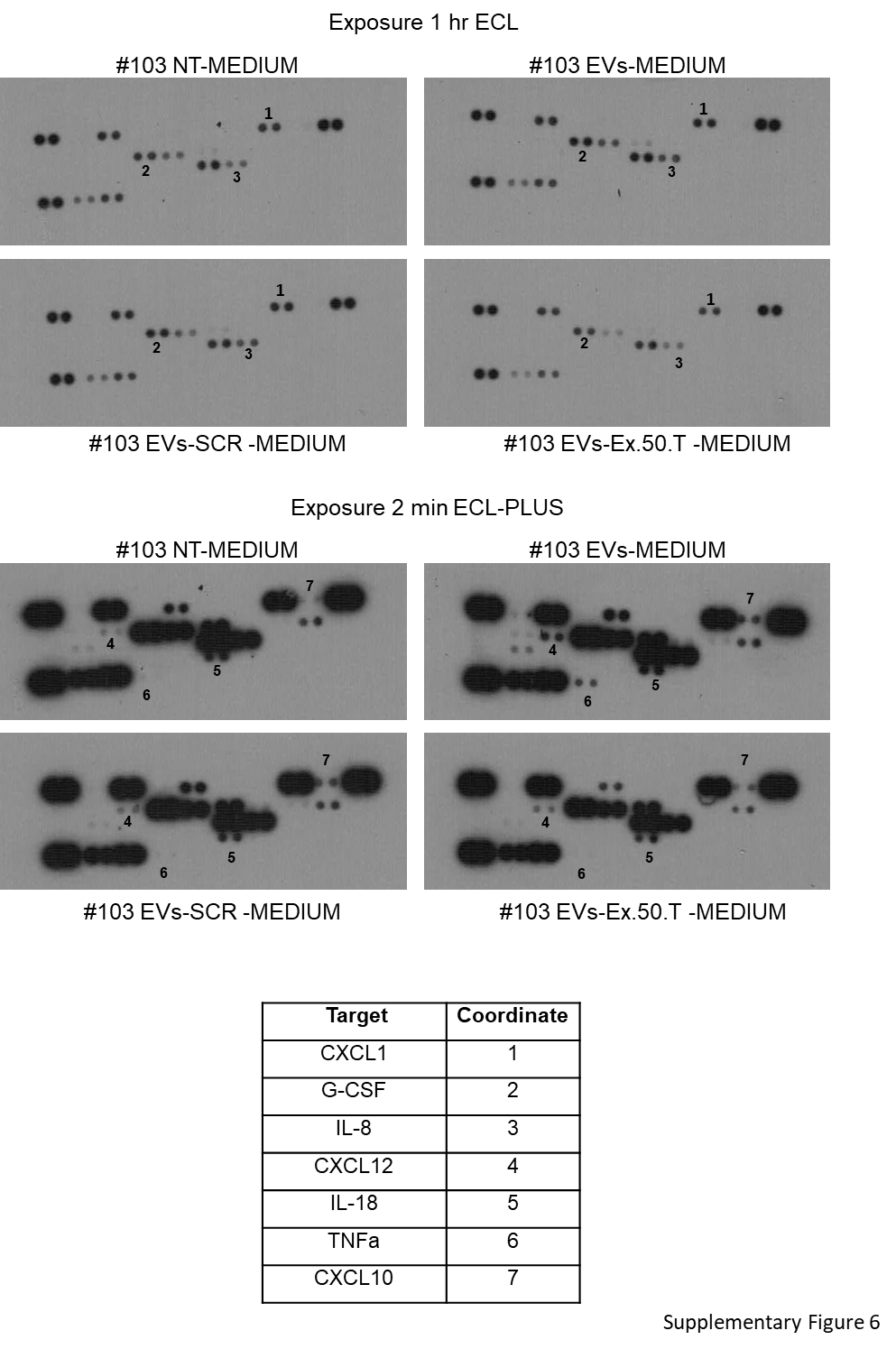


**Supplementary Figure 6**: Human Cytokine Array performed on media of NF#103 upon EVs, Control aptamer and ex.50.T treatment. Images were acquired after exposure of the membranes to 1hr exposure of ECL substrate (Upper Panel) or after 2 minutes of ECL plus (lower panel). In tables are reported the cytokine target and the number corresponding to the spot on the membranes


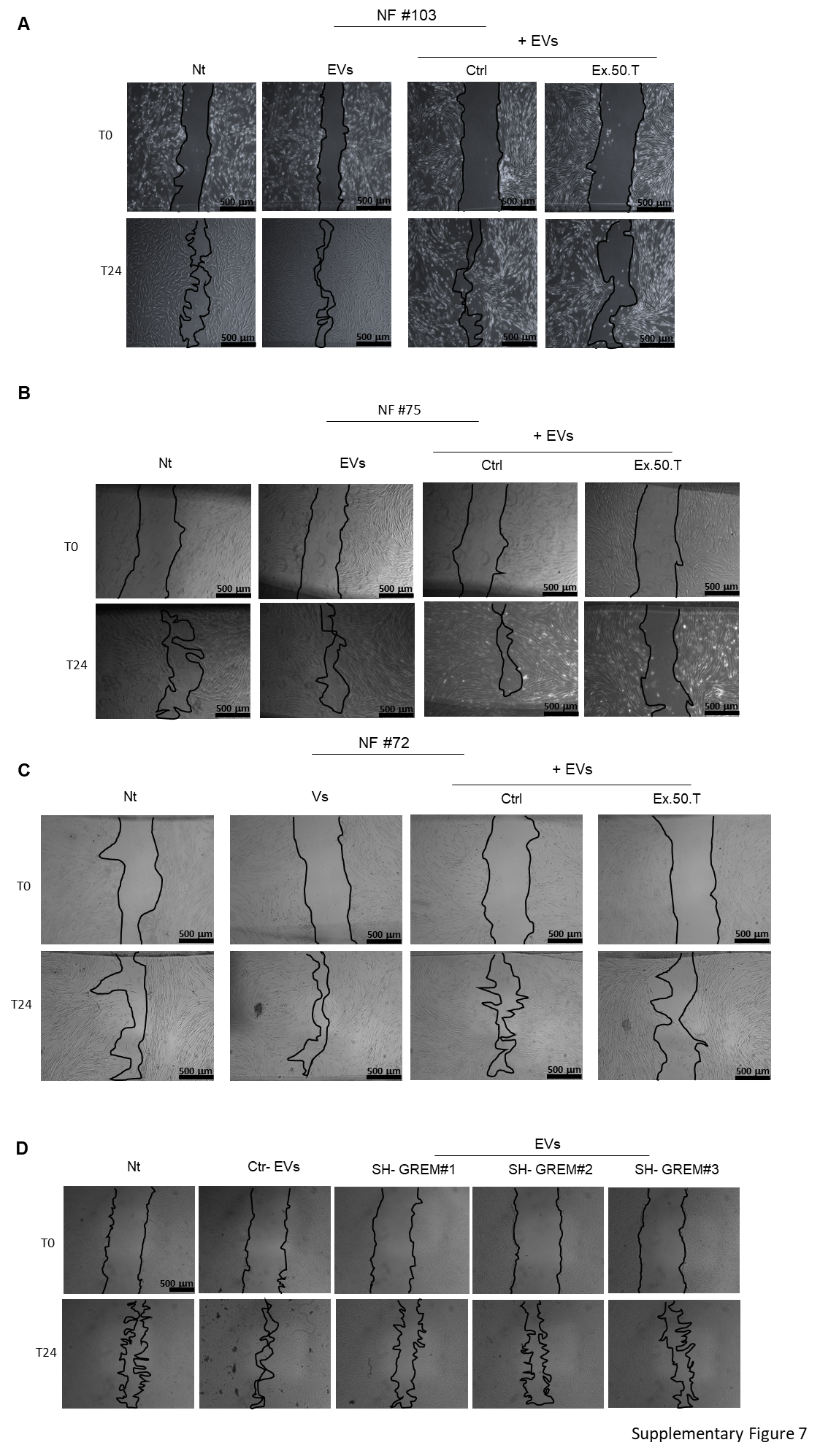


**Supplementary Figure 7**: Representative microscope images showing the area covered by the cells at 0 and 24 h after wound creation for NF103 **(A)**, NF#75 **(B)** and NF#72 **(C)** upon Evs, EVs and control aptamer and ex.50.T treatments. Images indicate a reduction of wound area when NFs were treated with BT549-derived EVs. **(D)** Representative microscope images showing the area covered by the cells at 0 and 24 h after wound creation for NFs treated with EVs from BT-549 stably transduced with SH-GREM1.
