# Supplementary material for: Ex.50.T aptamer impairs tumor–stroma cross-talk in breast cancer by targeting gremlin-1
Source: Cell Death Discov. 2025 Mar 11;11:94. doi: 10.1038/s41420-025-02363-6 (PMC11897156; doi:10.1038/s41420-025-02363-6)
Supplement: Supplementary file 2 — uncropped films Wb [file 41420_2025_2363_MOESM2_ESM.pptx]

## Slide 1
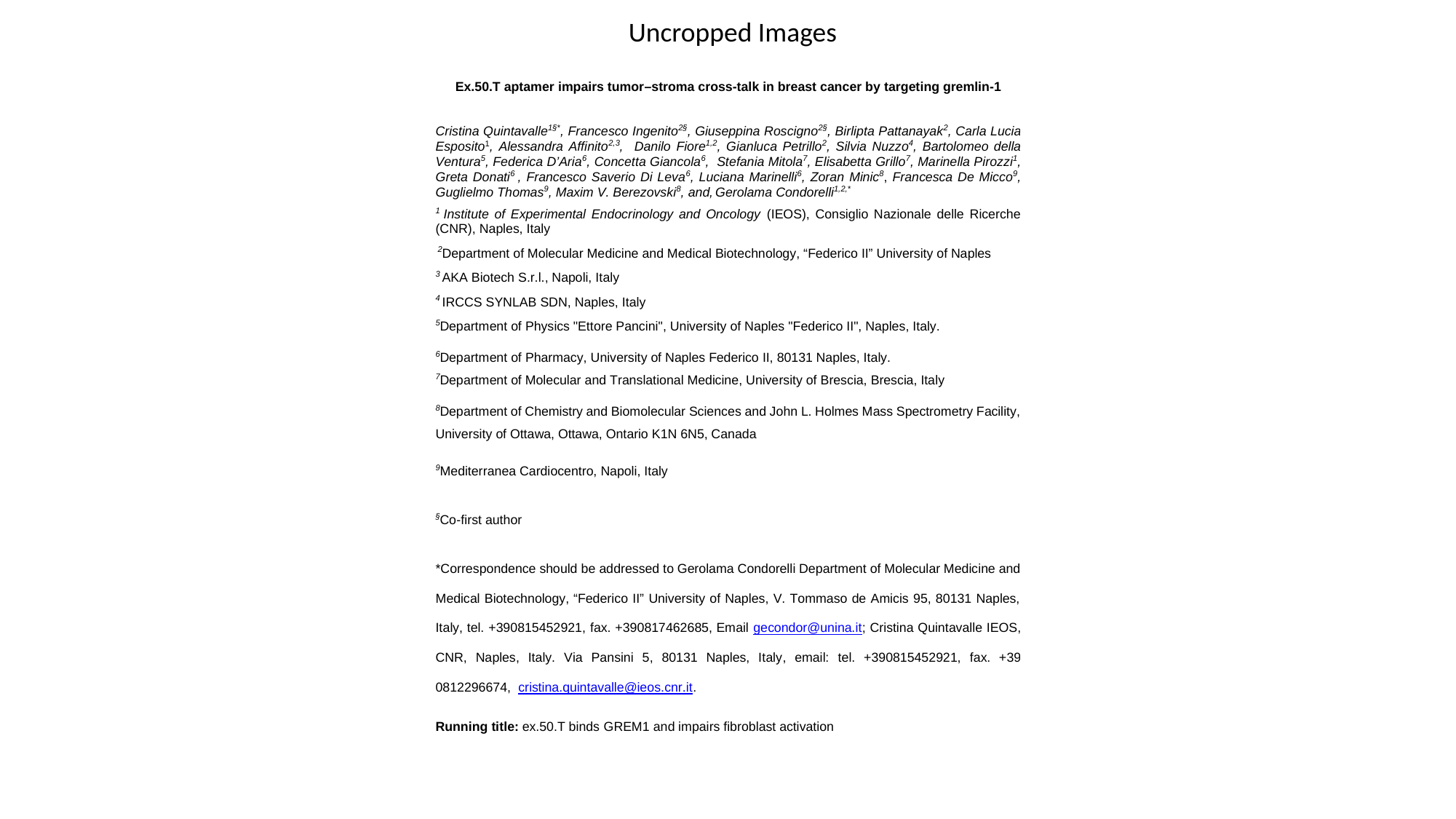

Uncropped Images

## Slide 2
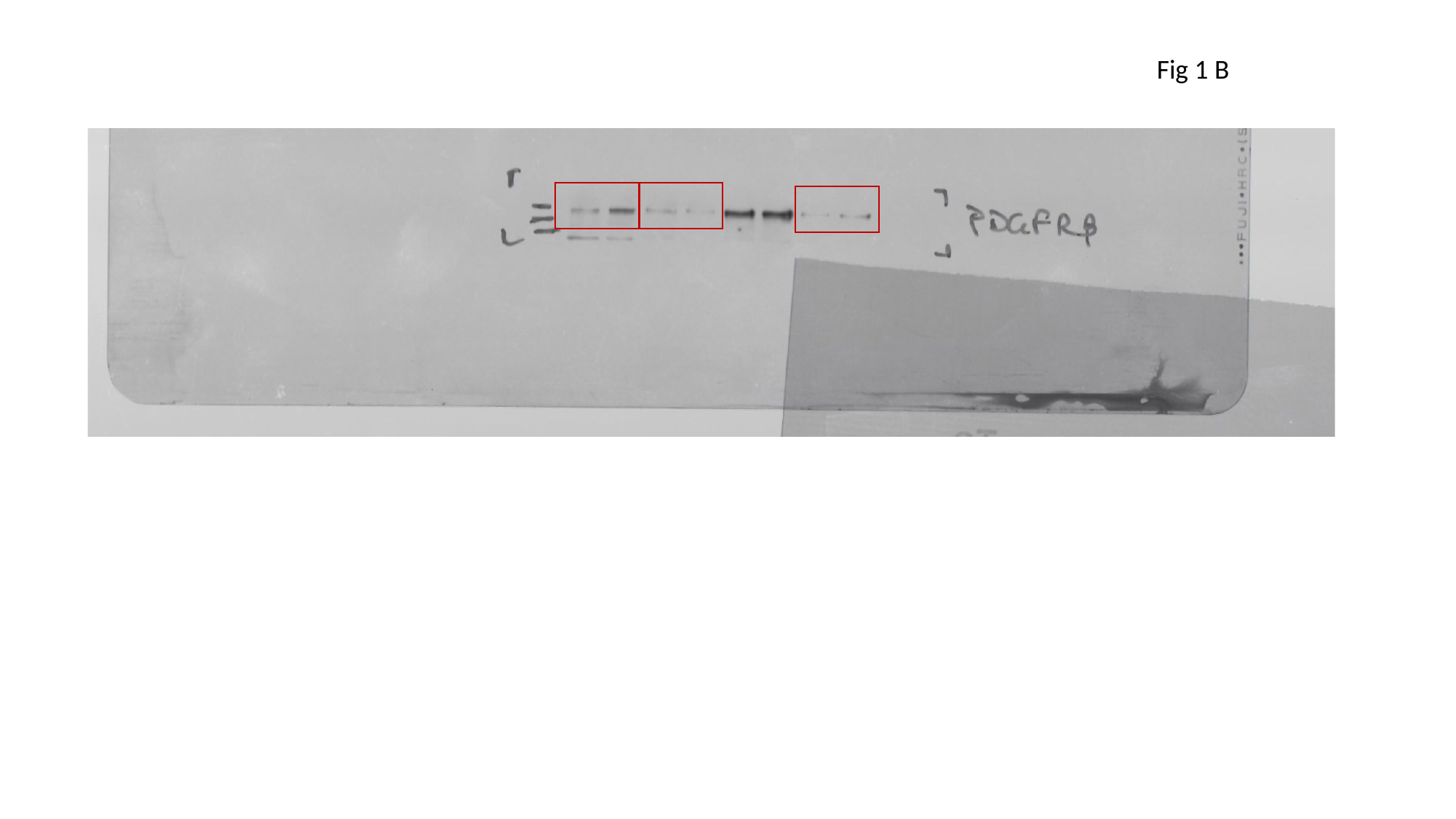

Fig 1 B

## Slide 3
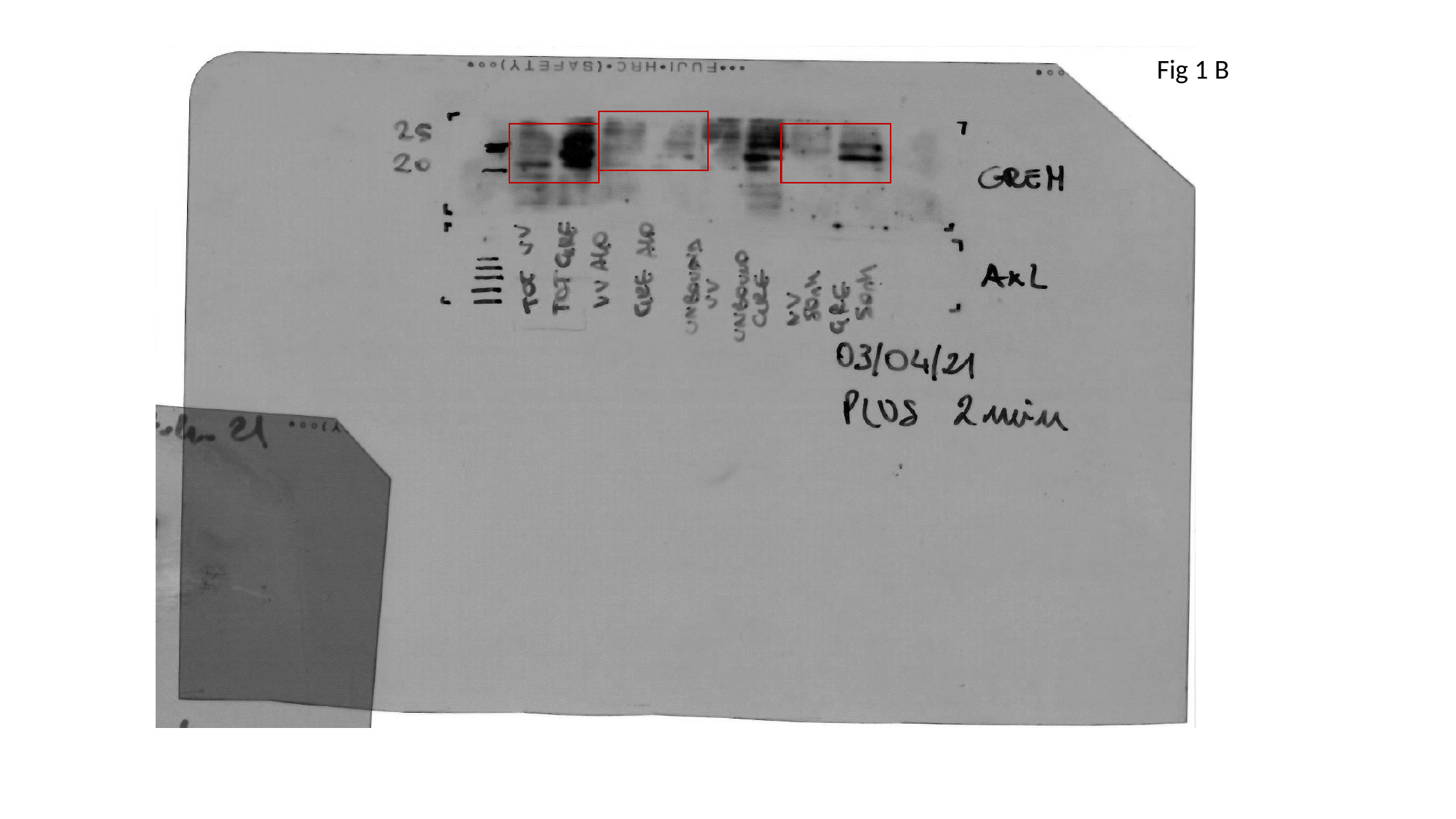

Fig 1 B

## Slide 4
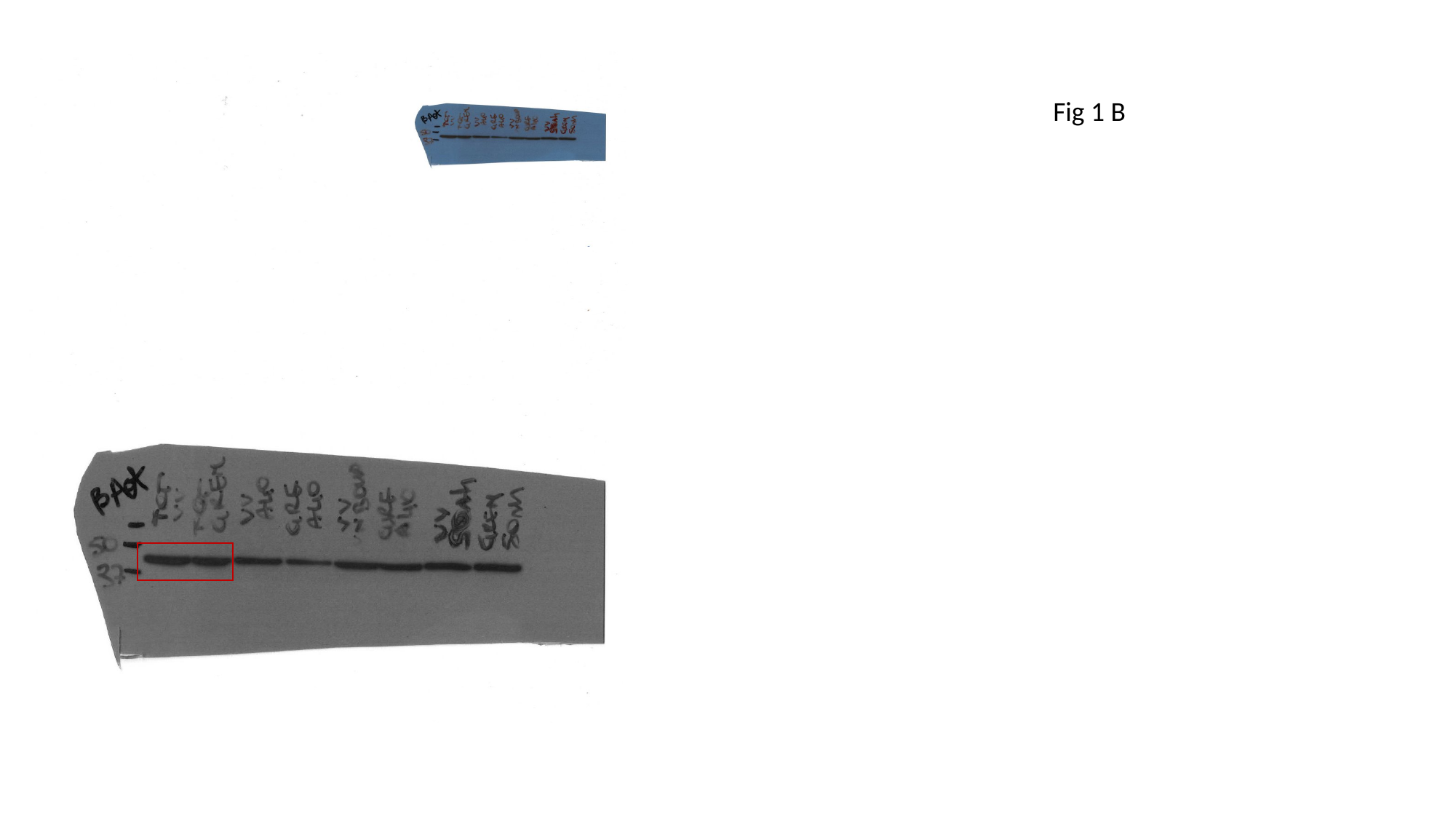

Fig 1 B

## Slide 5
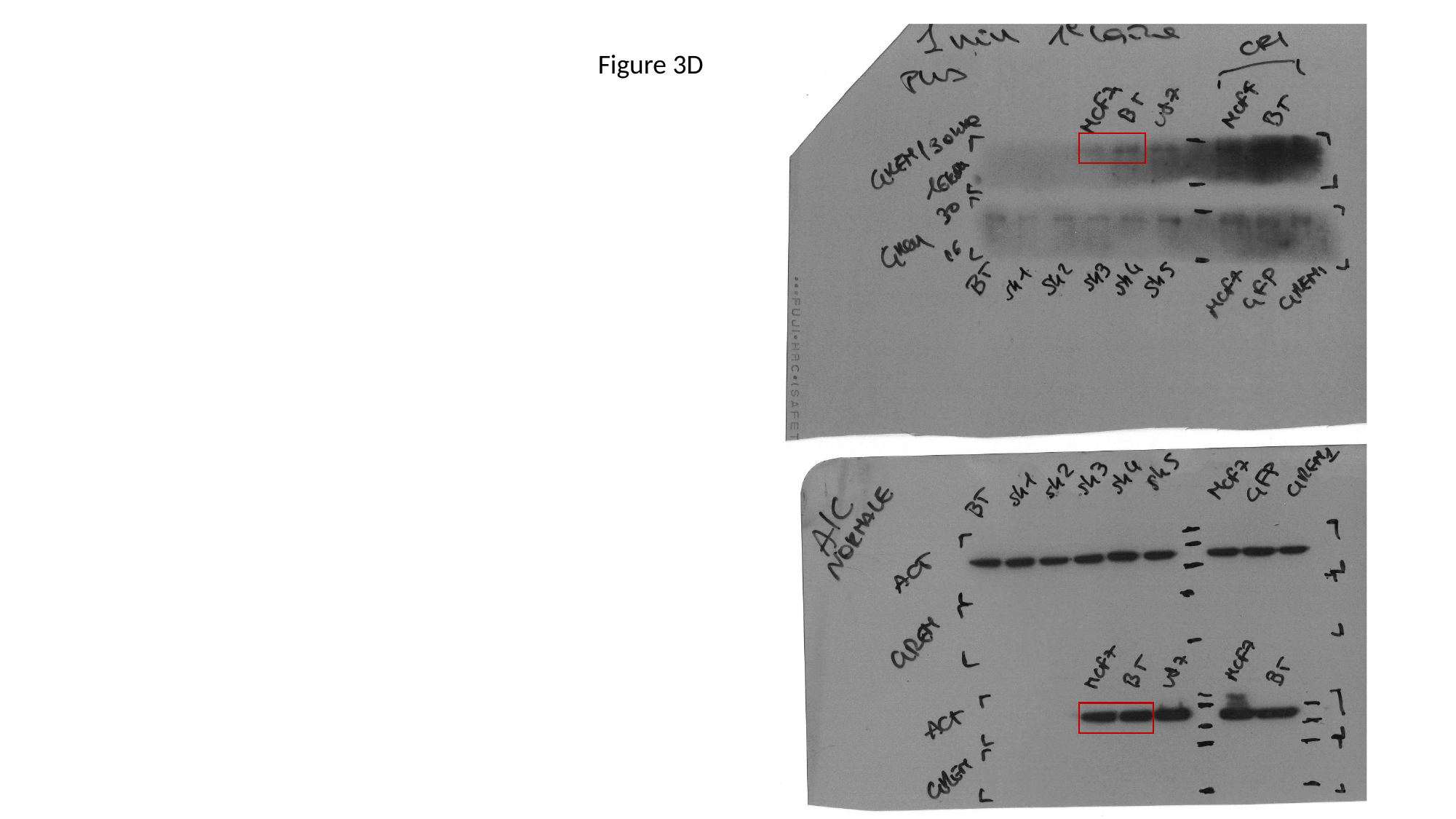

Figure 3D

## Slide 6
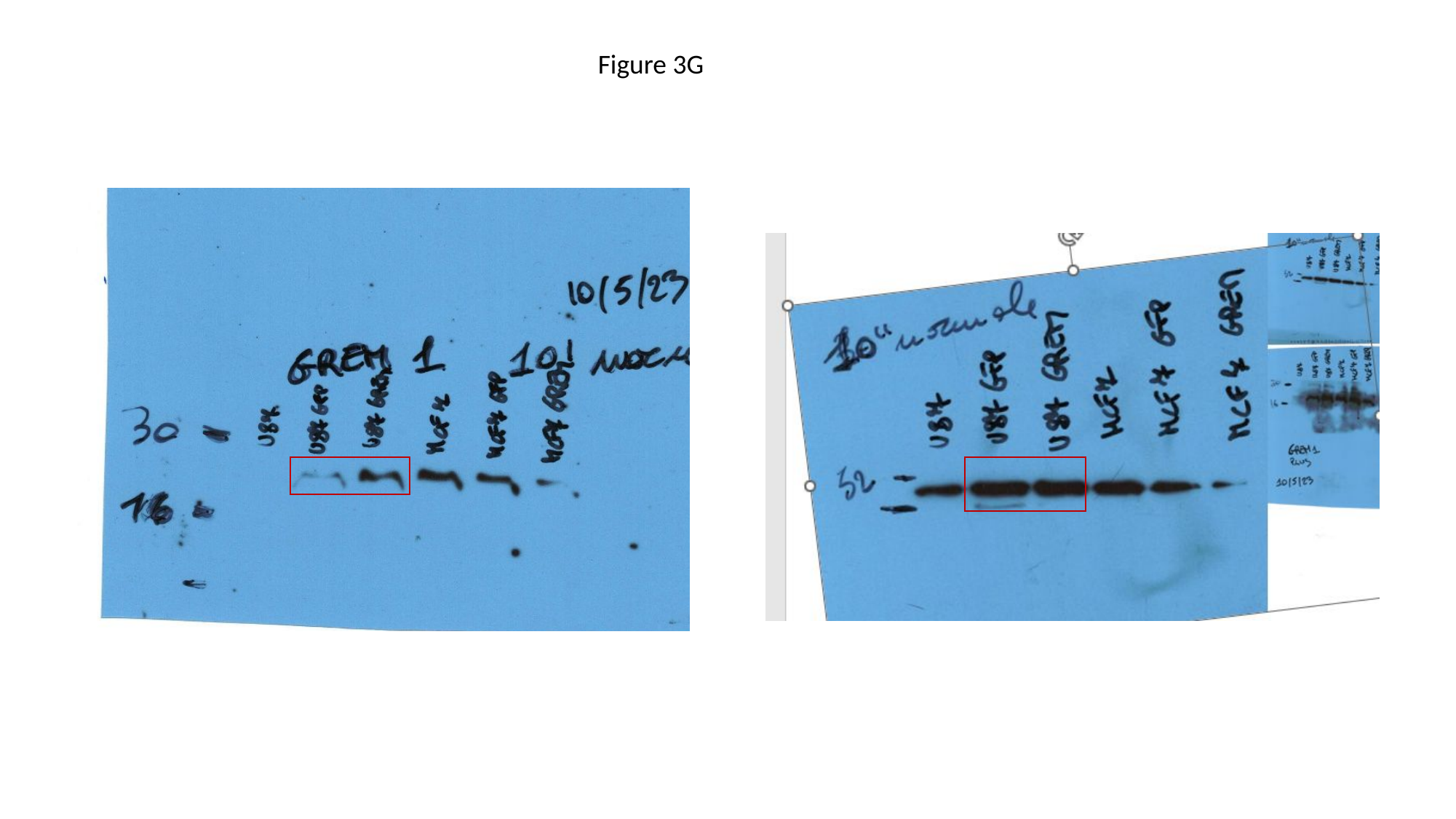

Figure 3G
#

## Slide 7
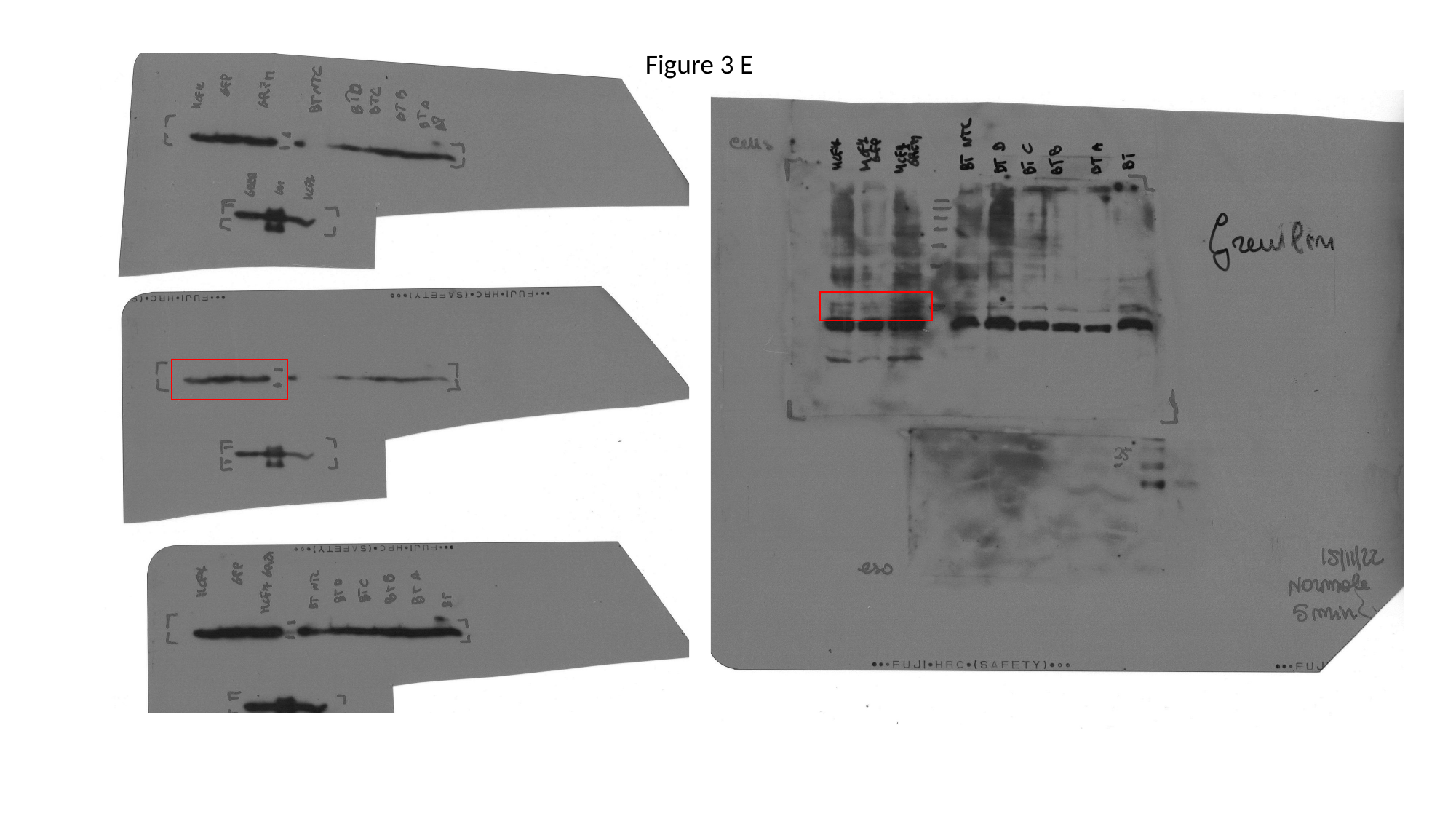

Figure 3 E
#

## Slide 8
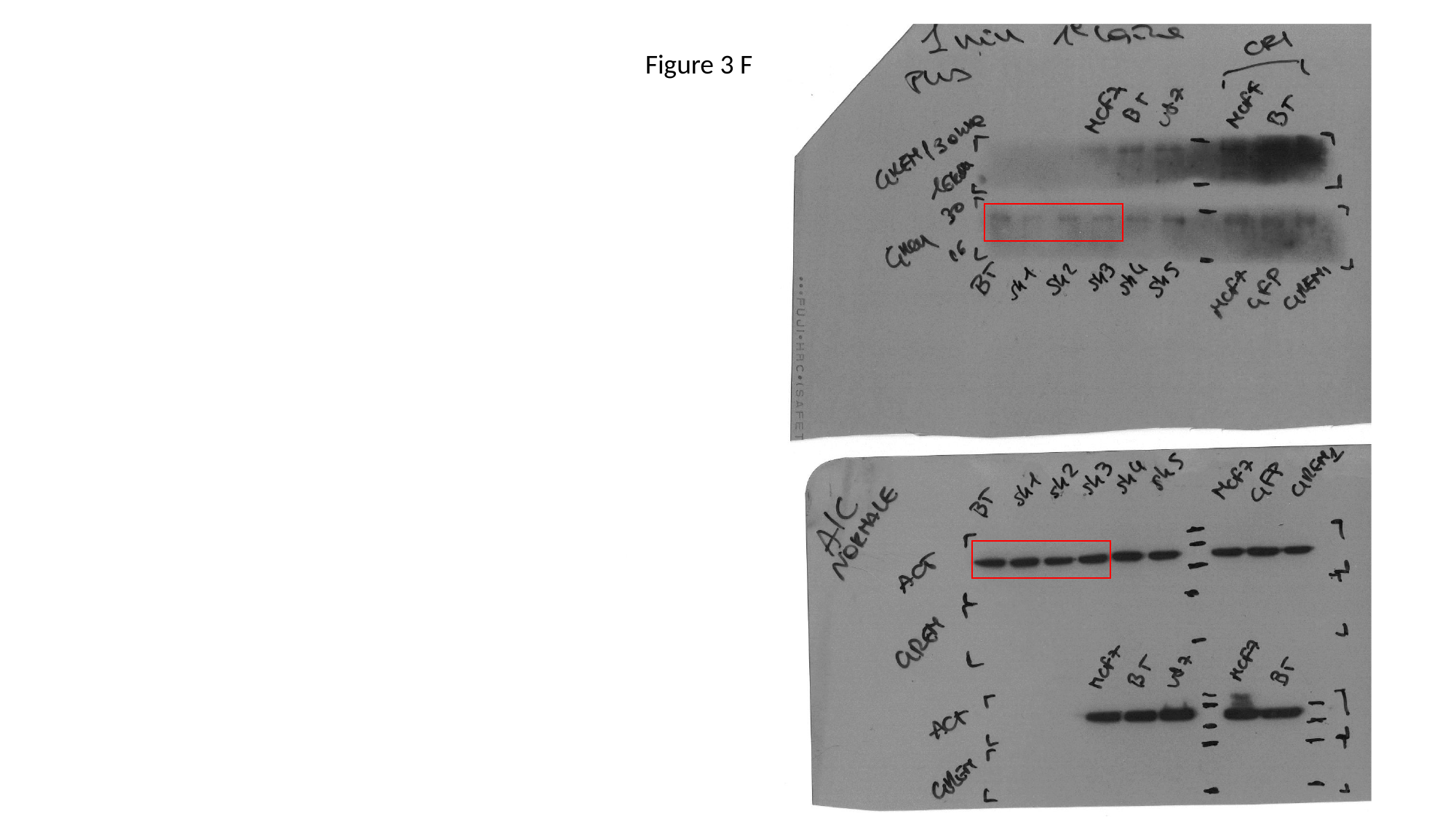

Figure 3 F

## Slide 9
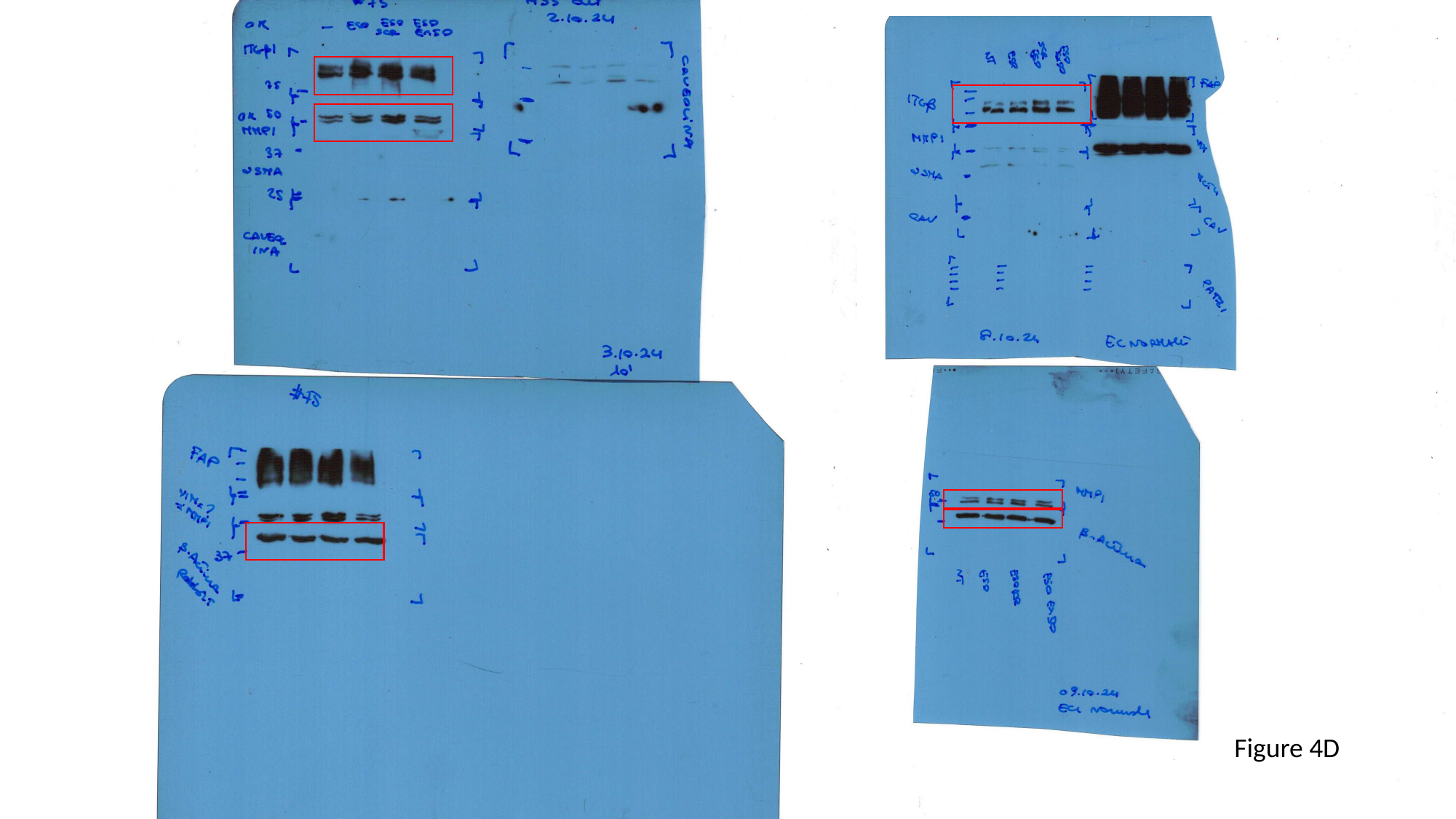

#
Figure 4D

## Slide 10
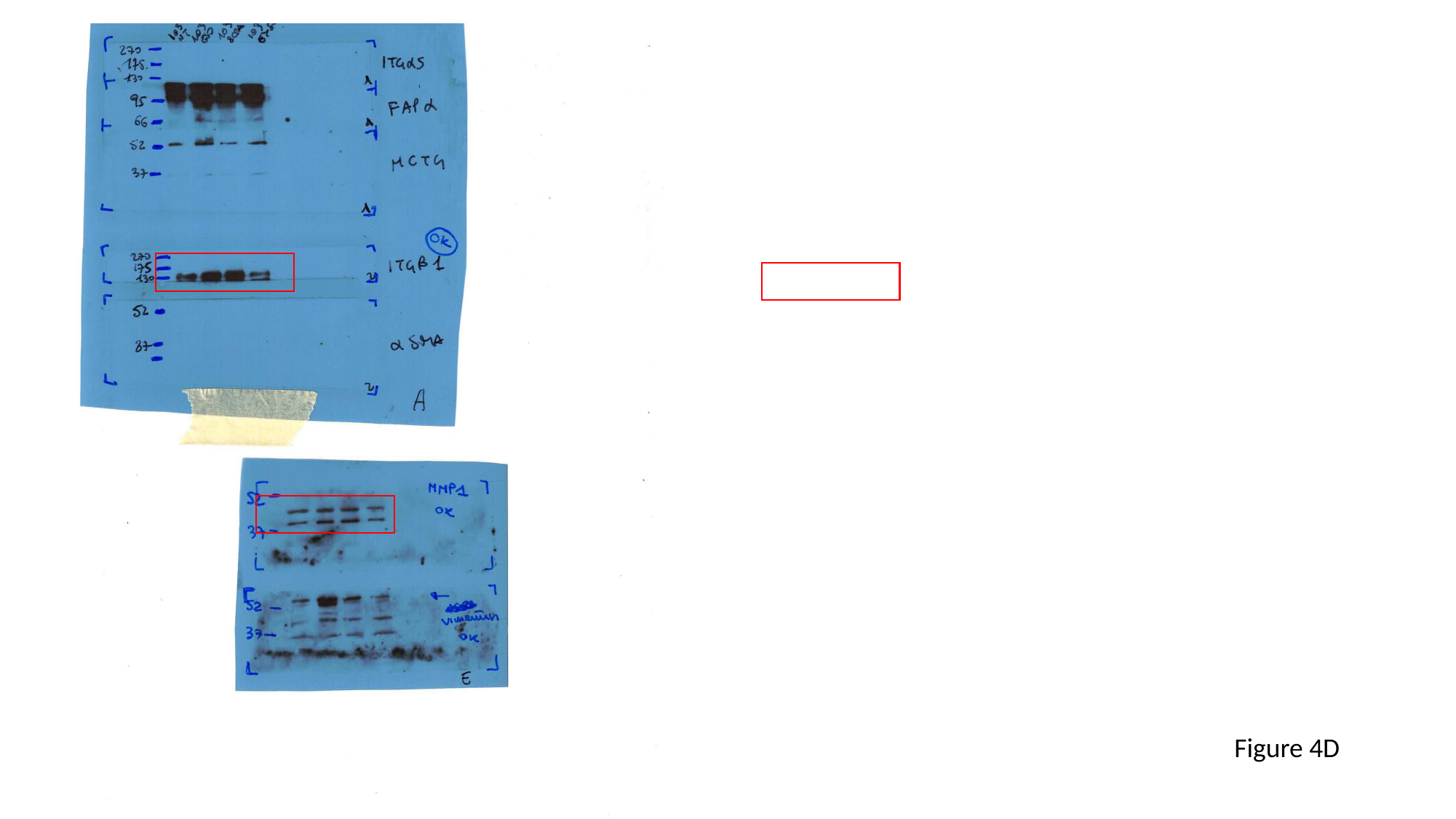

#
Figure 4D

## Slide 11
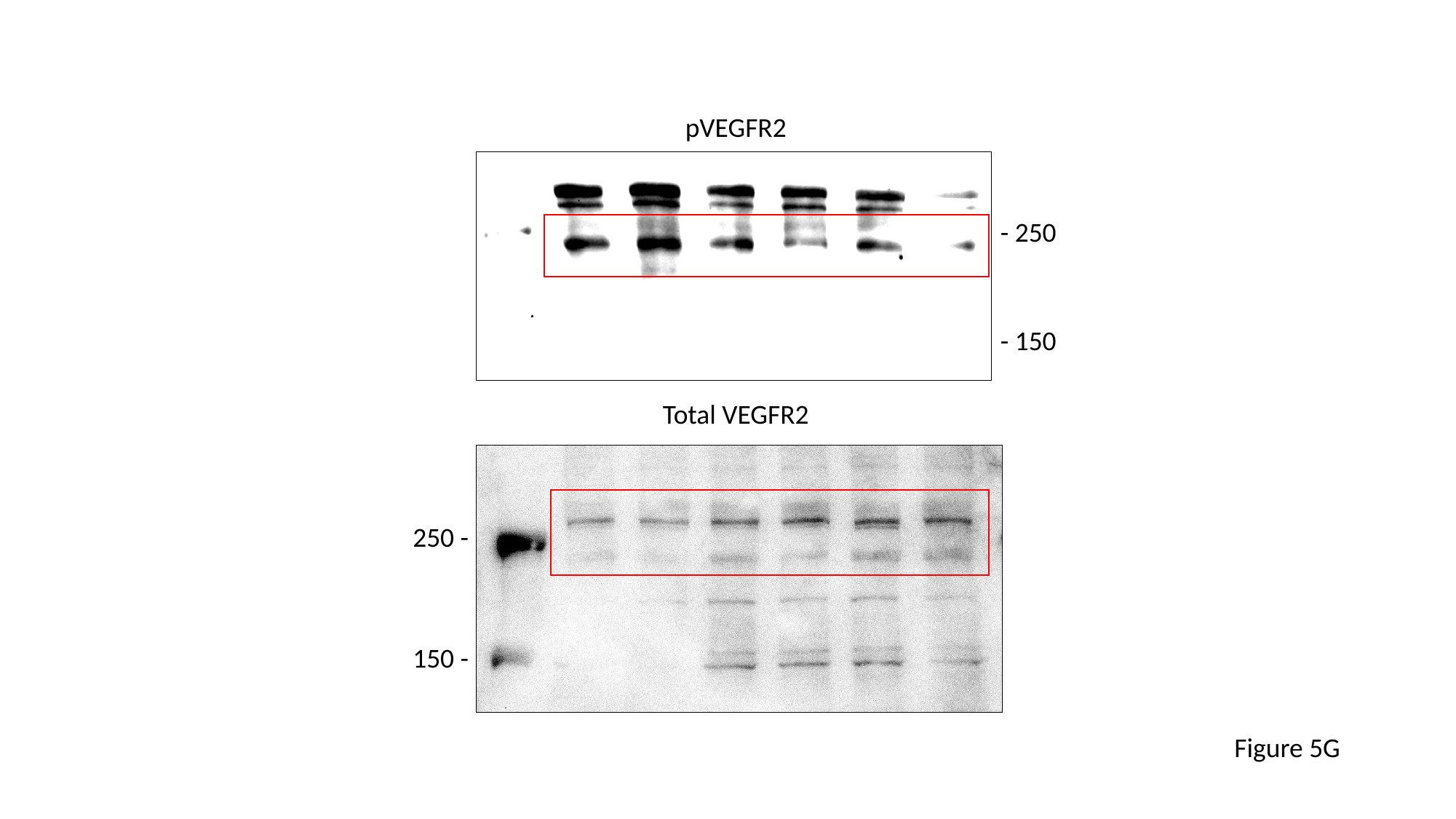

#
pVEGFR2
- 250
- 150
Total VEGFR2
250 -
150 -
Figure 5G

## Slide 12
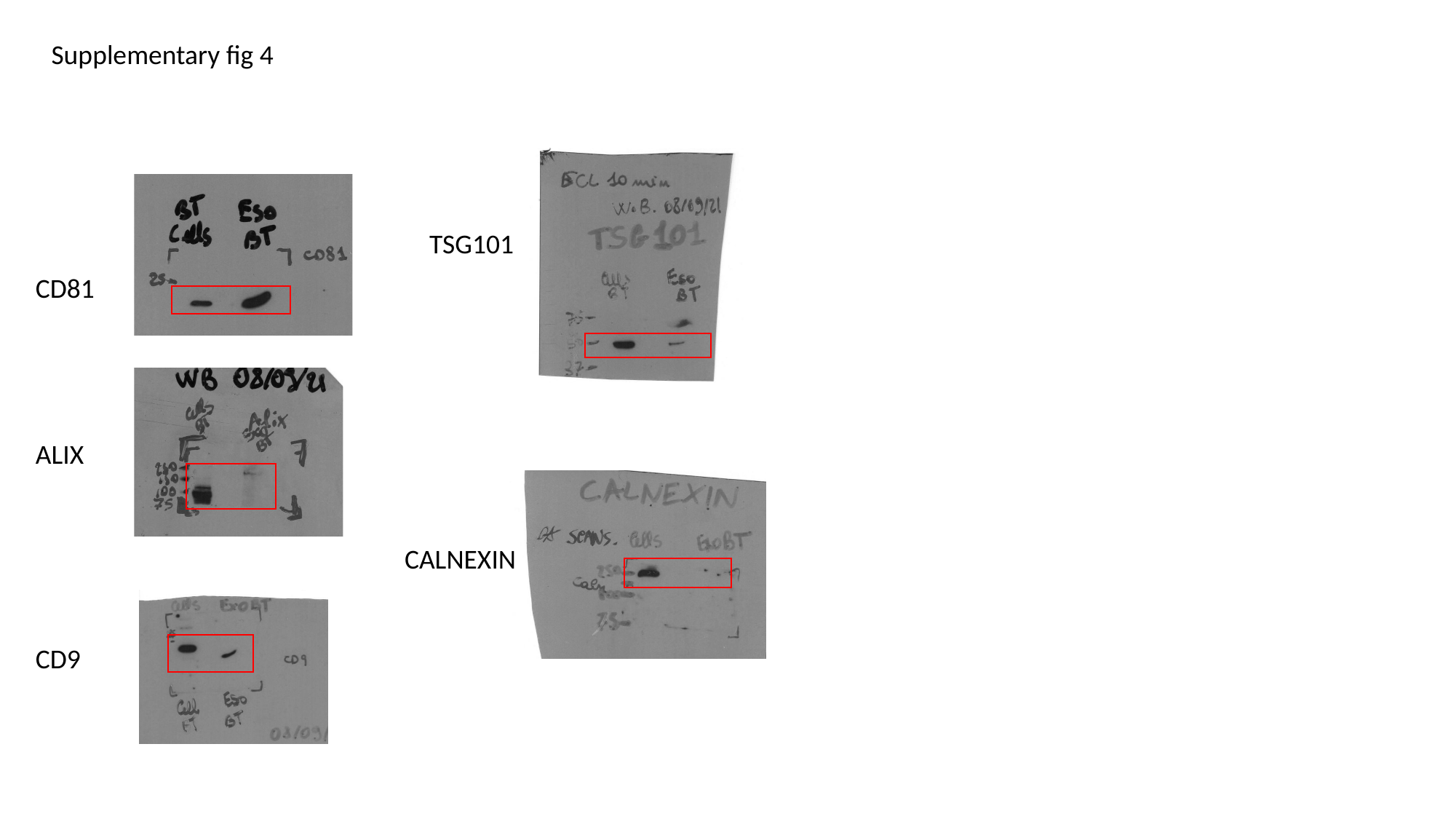

Supplementary fig 4
TSG101
CD81
ALIX
CALNEXIN
CD9

## Slide 13
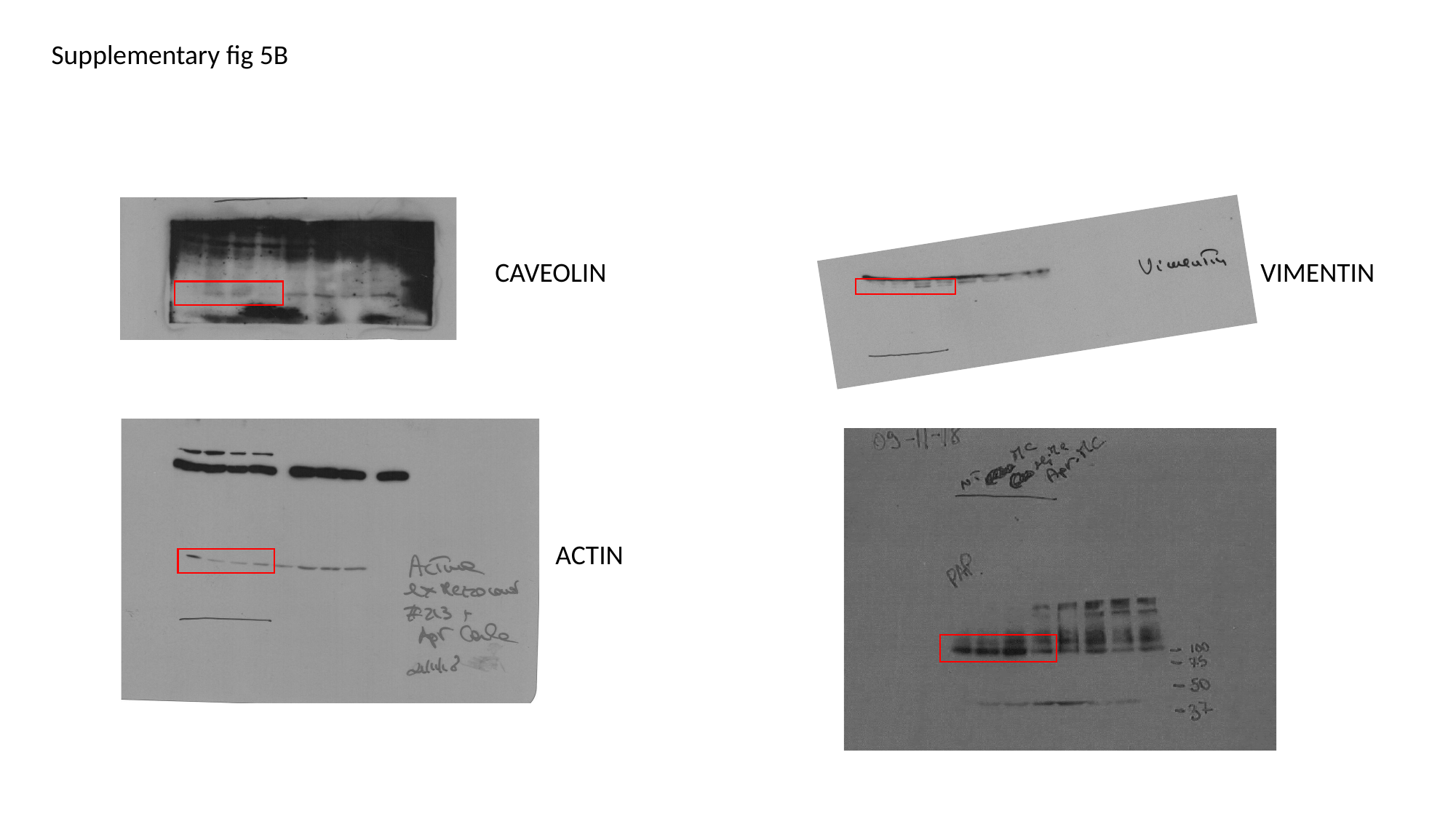

Supplementary fig 5B
CAVEOLIN
VIMENTIN
ACTIN
